# Downregulation of exosomal CLEC3B in hepatocellular carcinoma promotes metastasis and angiogenesis via AMPK and VEGF signals

**DOI:** 10.1186/s12964-019-0423-6

**Published:** 2019-09-02

**Authors:** Wenjuan Dai, Yilin Wang, Tianxiao Yang, Jing Wang, Weicheng Wu, Jianxin Gu

**Affiliations:** 1 Key Laboratory of Glycoconjugate Research Ministry of Health; Department of Biochemistry and Molecular Biology, School of Basic Medical Sciences, Fudan University, Shanghai, China; 2 The Key Laboratory of Public Health and Safety of Education Ministry, School of Public Health; School of Life Sciences and Human Phenome Institute, Fudan University, Shanghai, China; 3 Department of Hepatic Surgery, Fudan University Shanghai Cancer Center; Department of Oncology, Shanghai Medical College, Fudan University, Shanghai, China; 4 Shanghai Clinical Center for Endocrine and Metabolic Diseases, Shanghai Key Laboratory for Endocrine Tumors, Rui-Jin Hospital, Shanghai Jiao-Tong University School of Medicine, Shanghai, China

**Keywords:** CLEC3B, Exosomes, Hepatocellular carcinoma, Endothelial cells, VEGF, AMPK

## Abstract

**Background:**

C-Type Lectin Domain Family 3 Member B (CLEC3B), is down-regulated in serum and tumor tissues in different cancers including hepatocellular carcinoma (HCC). However, the functions of CLEC3B in HCC remains elucidated. The aim of this study is to analyze the roles of CLEC3B in HCC.

**Methods:**

The expression of genes was evaluated by immunohistochemistry, western blot, real-time PCR, enzyme-linked immunosorbent assays, and analysis on TCGA-LIHC database and gene expression omnibus. Transmission electron microscopy and immunofluorescence were applied to detect CLEC3B in exosomes. The function of exosomal CLEC3B in tumor progression were performed in vivo and in vitro.

**Results:**

We determined that down-regulated CLEC3B in HCC indicated a poor prognosis. Exosomes derived from HCC with down-regulated CLEC3B promoted migration, invasion, epithelial–mesenchymal transition of both tumor cells and endothelial cells (ECs). Moreover, the downregulation CLEC3B in exosomes suppressed VEGF secretion in both HCC cells and ECs, and eventually inhibited angiogenesis. Mechanistically, CLEC3B-mediated VEGF expression in tumor cells and ECs depends on the activation of AMPK signal pathway.

**Conclusion:**

This study demonstrates that CLEC3B acts as a novel independent prognostic factor, and CLEC3B in exosomes might be a potential therapeutic target for hepatocellular carcinoma.

**Graphical abstract:**

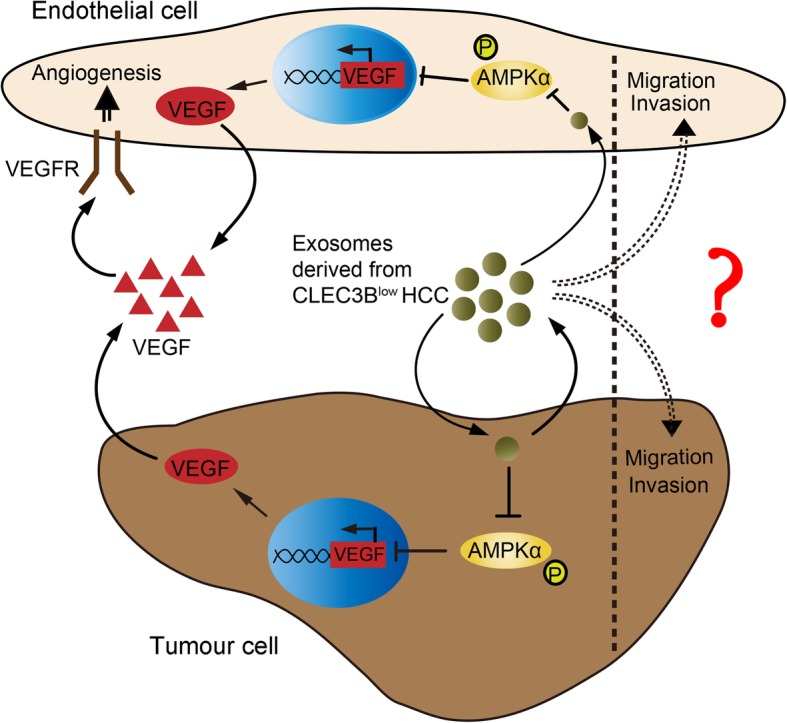

**Electronic supplementary material:**

The online version of this article (10.1186/s12964-019-0423-6) contains supplementary material, which is available to authorized users.

## Background

HCC is a highly vascularized solid tumor with rapid growth rate and poor prognosis [1, 2]. Angiogenesis, the process known as new vessels developing from pre-existing vessels, is the major mechanism for the growth and expansion of vascular network, which is essential for HCC growth and metastasis [3–5]. Angiogenesis mostly results from the secretion of several vascular endothelial growth factors (VEGFs) from tumor cells [6]. Among these VEGFs, VEGFA (also referred to VEGF) targets VEGFR2 on endothelial cells (ECs), and activates different signaling pathways, which directly regulates the proliferation, survival, migration and invasion of ECs, forms a lattice network and increases the permeability of existing blood vessels for the migration of ECs via remodeling the extracellular matrix, enhancing chemotaxis and homing of ECs, and finally promotes angiogenesis [7–9]. VEGF secreted by tumor cells also functions in an autocrine pattern, facilitates an epithelial–mesenchymal transition (EMT) phenotype for tumor cells, and promotes the stemness of tumor cells [10].

Besides VEGF, some other factors including secondary metabolites and exosomes were also identified to regulate angiogenesis [11, 12]. Exosomes are 30–100 nm extracellular vesicles enriched in endosome-derived components, secreted by living cells both in physiological and pathological conditions [13]. Exosomes carry content from host cells to change physiological feature of the recipient cells in microenvironment [14]. Activated biomolecules, like DNA, RNA and protein, are protected their biological activity from degradation in lipid bilayers and are delivered into target cells via endocytosis or membrane fusion. By modifying physiological state of tumor cells and cells related to tumors, tumor-derived exosomes (TDEs) regulate different aspects in tumor progression. TDEs transfer signals between tumor and surroundings to activate proliferative pathways, suppress tumor-associated immune reaction, initiate pre-metastatic sites and induce angiogenesis [15, 16]. It was reported that the highly expressed soluble E-cadherin is rich in exosomes from ovarian cancer and would induce sequential activation of β-catenin and NFκB signaling in ECs and eventually led to angiogenesis [12]. Abundant miR-25-3p in TDEs target KLF2 and KLF4 to influence the expression of VEGFR2 and EMT-associated genes in ECs, which would induce vascular permeability and angiogenesis [17].

Exosomes trigger different signals when sticking to target cells, including AMP-activated protein kinase (AMPK) signaling pathway. AMPK is a hetero-trimeric kinase modulated by cellular concentration of ATP, ADP and AMP. Changes in ATP-to-ADP ratio or ATP-to-AMP ratio resulting from changes in energy availability will activate AMPK through allosteric mechanism to stimulate kinase activity. In addition, AMPK would also be activated by the raise of cAMP [18]. AMPK is major mediator in cellular energy and metabolic control, and it is de-regulated in various disease, like diabetes and cancers [19]. Multiple findings have identified that AMPK acts as tumor suppressor in vivo and a widespread mechanism of downregulating AMPK signaling during tumorigenesis. Loss-of-function mutation or deletion of Lkb1/Stk11 will decrease AMPK signaling [19–21]. AMPK can activate TET2 at serine 99 to maintain it or inhibit mTOR pathway to impede tumor proliferation and metastasis and induce apoptosis [22–24].

C-Type Lectin Domain Family 3 Member B (CLEC3B) is a transmembrane Ca^2+^-binding protein, locating in cell plasma, extracellular matrix and exosomes. Downregulation of CLEC3B has been reported in various diseases. It was demonstrated in CLEC3B-deficient mice that the absence of CLEC3B impeded the mineralization process in osteogenesis [25]. Meanwhile, in patients with Coronary artery disease (CAD), serum levels of CLEC3B is significantly decreased, suggesting its potential effects in delaying CAD progress [26]. CLEC3B also plays a vital role in neuroprotection in Parkinson’s disease via lessening neuron apoptosis, and was suggested as an effective biomarker or potential therapeutic target in treating Parkinson’s disease [27]. Recently, some studies revealed that CLEC3B is involved in human cancer progression as well. CLEC3B is downregulated in both serums and saliva of patients with primary and lymph-node metastasis oral squamous cell carcinoma [28]. It was also reported that the positive expression of CLEC3B in tumor tissues and serums referred to a more favorable outcome for ovarian cancer patients [29]. It also has been reported that CLEC3B is also reduced in HCC [30]. However, the mechanisms of CLEC3B has never been reported in HCC. In this study, we investigated the roles of CLEC3B playing in HCC.

## Materials and methods

### Patient samples

Usage of human pathological tissues and clinical data was approved by the Ethics Committee at the Shanghai Cancer Center of Fudan University (Shanghai, China) (Approval No. 050432–4-1212B). Written consent for all patients conformed to the ethical guidelines of the Helsinki Declaration. A total of 180 patients with primary HCC resected between 2010 and 2012 in the Department of Hepatic Surgery, Shanghai Cancer Center of Fudan University (Shanghai, China) were collected. The patients were excluded if they had other malignant tumor before, or histories of adjuvant or neo-adjuvant therapies including targeted therapies. Clinicopathological data, including age, gender, tumor size (longest diameter), tumor location, Borrmann classification, differentiation, and TNM stage, were collected from medical history for each patient. Tumor stage was reassessed according to the seventh edition of the American Joint Committee on Cancer TNM classification. OS was defined as the time from the date of surgery to the date of death or last visit.

### Animal studies

All animal experiments were approved by the ethics committee of Fudan University (Shanghai, China). Four to five-week-old male BALB/c mice were purchased from Shanghai Laboratory Animal Center of Chinese Academy Sciences, which were housed in a sterile room. All of the mice were randomly grouped (n = 6 in each group). To establish the nude mouse xenograft model, Huh7-luc cells (5 × 10^6^ cells were suspended in 0.1 ml PBS) were subcutaneously injected into the left armpit of nude mouse (SLAC, Shanghai, China). Tumor xenografts were harvested four weeks later. A part of the tumor xenografts were fixed in paraformaldehyde for 12 h and embedded in paraffin for IHC assay. For the orthotopic transplantation model, tumors from xenograft models were cut in PBS on the ice. The diameter of each fragment was 1 mm. The fragments were then transplanted into nude mice in the left lobes of the liver. Bioluminescent imaging was performed with an IVIS200 (Xenogen, Caliper, California, US) 10–15 min after intraperitoneal injection of luciferin (3 mg/mouse) (Promega, WI, US). The intensity of luciferase signals was quantified using ROI analysis.

### Cell lines

The human HCC cell lines SMMC-7721, SK-HEP-1, BEL-7402 (7402), MHCC-97H, HCC-LM3, PLC/PRF/5 and Huh-7 were obtained from Cell Bank of Type Culture Collection of Chinese Academy of Science (Shanghai, China), and cultured in Dulbecco’s modified Eagle medium, DMEM (Sigma-Aldrich, St Louis, MO, USA) supplemented with 10% fetal bovine serum (FBS, Gibco, Grand Island, NY, USA) 1% penicillin-streptomycin. Human umbilical vein endothelial cells (HUVEC), purchased from Sciencell (Carlsbad, California, US), was cultured with Endothelial Cell Medium (Sciencell).All the cells were cultured at 37 °C in a humidified atmosphere containing 5% CO2.

### Tumor microarrays (TMAs), immunohistochemistry (IHC) and evaluation

The tumor microarrays (TMAs) were constructed from formalin-fixed, paraffin embedded surgical specimens, and CLEC3B staining was performed with UltraVision Quanto Detection system (Thermo scientific, California, US) following the protocol recommended and hematoxylin was used for counterstaining. The images were obtained by Nikon eclipse Ti-s microscope (Tokyo, Japan) and assessed by two investigators who had no knowledge of the patients’ clinical data to exclude subjectivity. For IHC results assessment, a previous scoring method was used [31]. Composite expression score (CES) with full range from 0 to 12 was performed to show the staining intensity and frequency of positive cells.

### Immunofluorescence (IF)

Cells cultured on cover slips were fixed with 4% paraformaldehyde and then washed with PBS (pH 7.4) for 3 times, each time for 5 min. 0.5% Triton X-100 was added to permeabilize. Next, blocking buffer consisting of BSA (Absin, Shanghai, China), 0.1% Triton X-100 was added to each well at room temperature. Next, cells were incubated with the primary antibody in blocking buffer at 4 °C overnight before the incubation with Alexa Fluor 594 AffiniPure Goat Anti-Mouse or Rabbit IgG (H + L) (Jackson, Pennsylvania, US) for 1.5 h at room temperature. After DAPI staining, the observed under Confocal Laser Scanning Microscopy (Leica Microsystems, Wetzlar, Germany).

### Enzyme linked immunosorbent assay (ELISA)

ELISA was performed followed manufacturer instruction (R&D Systems, Minnesota, US). The supernatant was collected from cells**.** Assay Diluent RD1W and sample was added successively to each well, and was incubated at room temperature. Each well was aspirated and washed by Wash Buffer for three times. After the last wash, Human VEGF Conjugate was added to each well and incubated at room temperature. Each well was aspirated and washed by Wash Buffer for three times. Substrate Solution was then added and protected from light, followed by adding Stop Solution and gently tapping the plate to ensure thorough mixing. The optical density was determined by a microplate reader set to 450 nm and readings at 450 nm was subtracted from the readings at 540 nm or 570 nm. The quantity of VEGF was calculated by standard curves. Each sample has 3 repeats and each assay was repeat 3 times.

### Migration and invasion assay

The migration and invasion of cells were assessed on Corning 12-well plates with transwell insert containing 8-μm pore filters (Millipore, Hessen state, Germany). Cells were seeded into each transwell inserts. For invasion assays, the upper of transwell chamber was coated with Matrigel Basement Membrane Matrix (Corning, New York, US) for 3 h in 37 °C. Cells were suspended in cell cultural medium or cellular supernatant and seeded into upper chambers. And cell cultural medium supplemented with serum were added into the bottom wells of the system. Migration or invasion of cells were determined 36 h or 48 h later. Both migratory and invasive cells were washed three times with ice-cold PBS and then fixed with 4% paraformaldehyde for 15 min. 0.1% crystal violet (Beyotime Institute of Biotechnology, Jiangsu, China) was used to stained the cells. Cells on the upper side of the filers were removed and the filters were washed with PBS for three times. Cells were observed by a microscope and counted. Each experiment was repeated 3 times.

### Wound healing assay

Cells were seeded in 6-well plates for wound healing assay. After cell attachment, cells were gently scratched in a straight line on the cell layer with pipet tips. Images of cells were taken at different time points. The cell scratch area was measured by ImageJ. Each experiment was repeated 3 times.

### Tube formation assay

In vitro tube formation analysis was evaluated on BD Matrigel Basement Membrane Matrix (Corning, New York, US) in a 48-well plate. Cells were cultured on the Matrigel, and treated with exosomes or supernatant derived from tumor cells. The total tube area was quantified as mean pixel density obtained from image analysis of 4 random microscopic fields using Image J software.

### Transmission Electron microscopy

For electron microscopy analysis, exosomes were prepared, fixed with 4% paraformaldehyde and 1% glutaraldehyde in 0.1 M phosphate buffer, pH 7.4, at incubation temperature, and placed in a refrigerator to lower temperature to 4 °C. The samples were adsorbed to a 400-mesh carbon-coated grid and immersed in 2% phosphotungstic acid solution (pH 7.0) for 30 s. The samples were observed by transmission electron microscope (FEI Company, Oregon, US) at an appropriate acceleration voltage.

### Isolation of exosomes

Exosomes were isolated with ExoQuick-TC™ Exosome Precipitation Solution (System Bioscience, California, US) following the manufacturer’s protocol. Briefly, the cell culture supernatants were harvested and then centrifuged at 3000 g for 15 min to remove cells and cell debris. Exosome Precipitation Solution was added to the supernatants, mixed completely. The mixture was centrifuged and supernatants were aspirated, and the exosome pellet was resuspended for further application. Exosomes derived from tumor cells with CLEC3B overexpression were named Exo-3B, and exosomes derived from tumor cells with CLEC3B down-regulated were named Exo-3B-KD.

### TCGA-LIHC and GEO database

The databases applied in this study are publically available from the Cancer Genome Atlas - Liver Hepatocellular Carcinoma (TCGA-LIHC) and Gene Expression Omnibus (GEO) database (GSE14520, GSE36376, GSE54236, GSE64041, GSE25097, GSE76427, GSE57958). For TCGA-LIHC database, gene expression dataset was downloaded from the TCGA-LIHC portal by using TCGA-Assembler software. The whole and 49 pairs of hepatocellular carcinoma/peritumor samples were analyzed. The relative mRNA expression was acquired from Gene Expression Omnibus (GEO).

### Plasmid

The cDNA encoding CLEC3B was achieved by PCR and cloned into the p3xFLAG-CMV-14 vector. The px260-CLEC3B was obtained as instruction. px260 plasmid was digested with Bbsl (Thermo scientific, Fastdigest Bpi I, FD1014) and a pair of oligos were annealed (Beyotime Biotechnology, Shanghai, China), which were cloned into the CRISPER array. The oligos is designed based on the target site sequence (30 bp) and needs to be flanked 0n the 3′ end by a 3 bp NGG PAM sequence, which is shown:

**Table:** 

gRNA	AAACGGCATTTACAATCTTCTTGGGCTTCTGGGTGT
tracrRNA	TAAAACACCCAGAAGCCCAAGAAGATTGTAAATGCC

### Real-time PCR

Total RNA was purified from stomach tissues or cancer cells using TRIzol (Invitrogen, Carlsbad, CA, USA) according to the manufacturer’s instructions. The RNA was then applied for reverse transcription and quantitative PCR using a Takara RNA PCR Kit and SYBR Premix Ex Taq (Takara, Tokyo, Japan) in accordance with the manufacturer’s instructions. GAPDH was used as an internal control. Primer is shown:

**Table:** 

CLEC3B RT P1	CCCAGACGAAGACCTTCCAC
CLEC3B RT P2	CGCAGGTACTCATACAGGGC
E-cadherin RT P1	CGAGAGCTACACGTTCACGG
E-cadherin RT P2	GGGTGTCGAGGGAAAAATAGG
GAPDH RT P1	GAGTCAACGGATTTGGTCGT
GAPDH RT P2	TTGATTTTGGAGGGATCTCG
HIF-1α RT P1	GAACGTCGAAAAGAAAAGTCTCG
HIF-1α RT P2	CCTTATCAAGATGCGAACTCACA
N-cadherin RT P1	TCAGGCGTCTGTAGAGGCTT
N-cadherin RT P2	ATGCACATCCTTCGATAAGACTG
Slug RT P1	CGAACTGGACACACATACAGTG
Slug RT P2	CTGAGGATCTCTGGTTGTGGT
Snai1 RT P1	TCGGAAGCCTAACTACAGCGA
Snai1 RT P2	AGATGAGCATTGGCAGCGAG
VEGF RT P1	AGGGCAGAATCATCACGAAGT
VEGF RT P2	AGGGTCTCGATTGGATGGCA
Vimentin RT P1	GACGCCATCAACACCGAGTT
Vimentin RT P2	CTTTGTCGTTGGTTAGCTGGT
ZO-1 RT P1	CAACATACAGTGACGCTTCACA
ZO-1 RT P2	CACTATTGACGTTTCCCCACTC
β-catenin RT P1	AAAGCGGCTGTTAGTCACTGG
β-catenin RT P2	CGAGTCATTGCATACTGTCCAT

### Western blotting

Protein in cell lysates were separated by SDS-polyacrylamide gel electrophoresis and transferred onto polyvinylidene difluoride membranes (Millipore, Hessen state, Germany), followed by incubated with primary antibodies against CLEC3B (Abcam, Massachusetts, US), HIF-1α, VEGF (Proteintech, Chicago, US), phospho-AMPKα, AMPKα, CD31, CD34 and EMT associated molecules (Cell Signaling Technology, Danvers, US) overnight in 4 °C. Next day, the membranes were subsequently incubated with horseradish peroxidase (HRP)-conjugated secondary antibody (Santa Cruz Biotechnology, California, US). The immunoreactive protein were visualized by using enhanced chemiluminescence detection kit (Tiangen Biotech, Beijing, China) and image analyzer ImageQuant LAS 4000 (GE Healthcare, Abingdon, UK). To ensure equal loading of plasma protein, the membranes were stained with 0.2% Ponceau S (Sinopharm Chemical Reagent, Shanghai, China) to ensure equal loading of the proteins.

### Statistical analysis

Statistical analyses were performed with SPSS 19 (SPSS Inc., Chicago, IL), GraphPad Prism 5 (GraphPad software, La Jolla, CA, USA), Stata 12.0 (Stata CorpLP, College Station TX, USA) and R software version 3.4.2 with the “rms” package (R Foundation for Statistical Computing, Vienna, Austria). ROC curve analysis was used to determine the optimal cutoff value for CES and compare the prognostic accuracy for combination model. Pearson’s chi-squared test and Fisher’s exact test were applied for categorical variables; continuous variables were analyzed by the Student’s t test. Survival and univariate analysis were determined by Kaplan-Meier analysis, and the significance of the difference between curves was calculated with the log-rank test. The Cox proportional hazards regression model was applied to perform multivariate analysis. Nomogram was generated by R software with “rms” package. The prognostic accuracy was also measured by calculating the C-index and AIC. All statistical analyses were two sided, and P value < 0.05 was considered statistically significant. Pearson’s chi-squared test and Fisher’s exact test were applied for categorical variables, and continuous variables were analyzed by the Student’s t test. Multiple comparison between the groups was performed using S-N-K method. All statistical analyses were two sided, and P value < 0.05 was considered statistically significant.

## Results

### Down-regulated CLEC3B is a predictor of poor prognosis in HCC patients

**Fig. 1:**
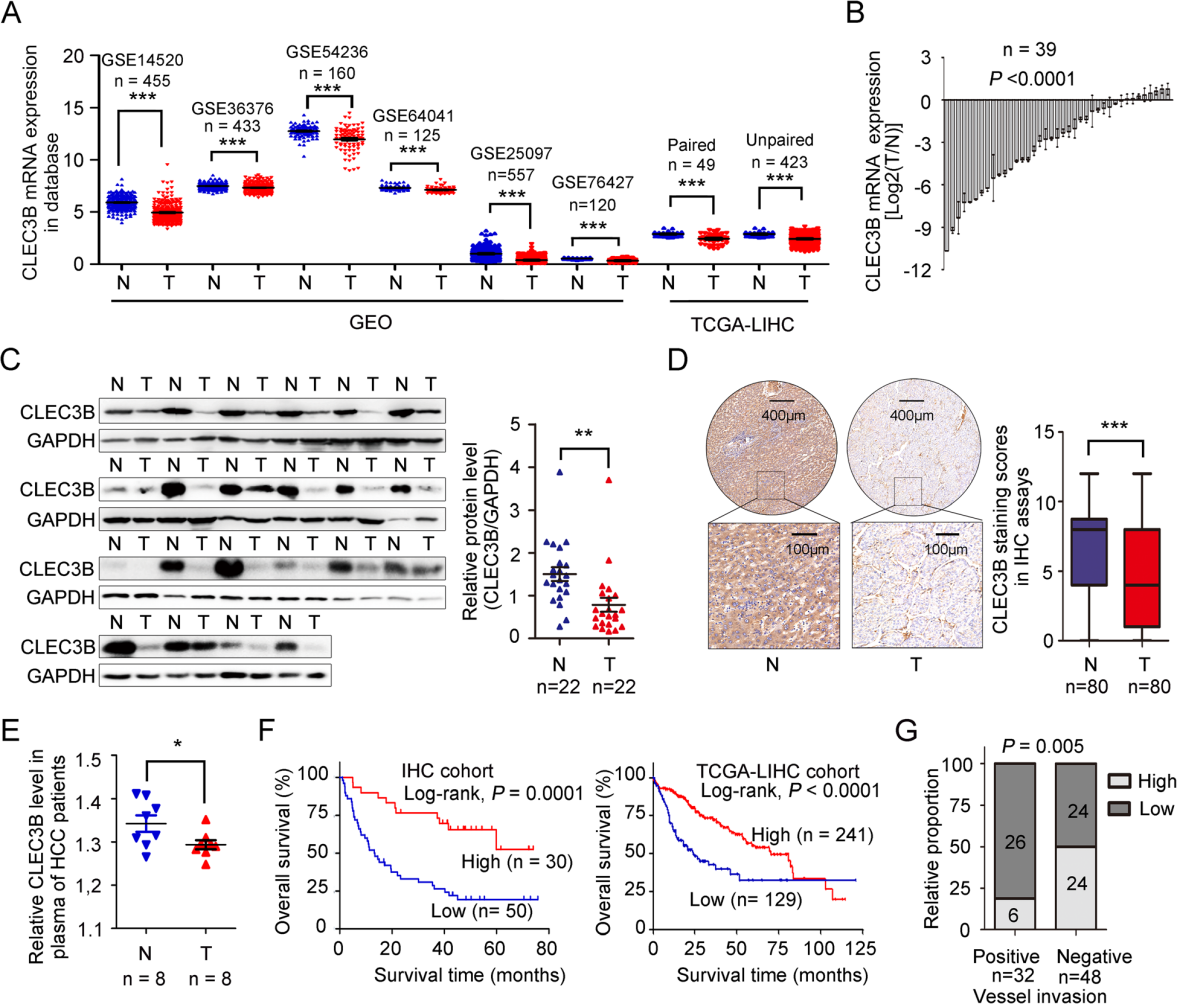
CLEC3B expression was down-regulated in HCC. **a** The mRNA levels of CLEC3B in tumor tissues (T) and non-tumor tissues (N) of hepatocellular carcinoma patients in Gene Expression Omnibus (GEO) database online, they are GSE14520 (n = 455, *P* < 0.0001), GSE36376 (n = 433, *P* < 0.0001), GSE54236 (n = 160, *P* < 0.0001), GSE64041 (n = 125, *P* < 0.0001), GSE25097 (n = 557, *P* < 0.0001) and GSE76427 (n = 120, *P* < 0.0001), and TCGA-LIHC cohort (log_10_), paired (*P* < 0.0001) and unpaired (*P* < 0.0001), from left to right. **b** The mRNA levels of 39 cases of hepatocellular carcinoma and adjacent non-tumor tissues were determined by RT-PCR (*P* < 0.0001). **c** The protein levels of 22 cases of hepatocellular carcinoma (T) and paired adjacent non-tumor (N) tissues were determined by western blot (left), and the statistical value of corresponding protein level of CLEC3B (right) (*P* = 0.0033). **d** The immunohistochemistry (IHC) results of representative high and low expression of CLEC3B in tumor and the statistical value of IHC of CLEC3B expression in hepatocellular carcinoma (T) and paired adjacent non-tumor (N) tissues (*P* < 0.0001). **e** Relative quantity of CLEC3B in plasma of the normal (N, n = 8) and HCC (T, n = 8) patients (*P* = 0.0425). **f** Overall survival in hepatocellular carcinoma patients (left, n = 80, *P* = 0.0001) and TCGA-LIHC cohort (right, n = 370, *P* < 0.0001), based on CLEC3B expression level, were calculated by Kaplan–Meier. **g** The relative proportion of patients with high CLEC3B expression increased from vessel metastatic patients (positive) to non-vessel metastatic patients (negative) (*P* = 0.005). *, *P* < 0.05; **, *P* < 0.01; ***, *P* < 0.001; n.s., not significant

To investigate whether CLEC3B expression is correlated to HCC development, we firstly analyzed the expression of CLEC3B in HCC (T) and peritumor samples (N) in 6 independent public datasets from Gene Expression Omnibus (GEO) database and TCGA-LIHC database, and proved that the mRNA levels of CLEC3B in these dataset was significantly down-regulated either (GSE14520 [32], GSE36376 [33], GSE54236 [34], GSE64041 [35], GSE25097 [36] and GSE76427 [37]), the same results as in paired and unpaired tumor and peritumor samples in TCGA-LIHC database (Fig. 1a). Then we detected the expression of CLEC3B in HCC tumor (T) and peritumor samples (N), and found that CLEC3B was also significantly down-regulated at both mRNA (39 pairs) and protein (22 pairs) levels in tumors in quantitative real-time PCR analysis and in western blot (Fig. 1b-c). Immunohistochemistry (IHC) staining assay was performed in tissue microarrays of 80 pairs of HCC samples to reveal that the protein levels of CLEC3B was significantly decreased in tumor tissues (Fig. 1d).

The down-regulation of CLEC3B in the serum of some patients with ovarian cancer or oral squamous cell carcinoma has been reported in previous studies [28, 29]. Similar to these results, we also detected decreased CLEC3B in serum of HCC patients (Fig. 1e and Additional file 1: Figure S1A), suggesting that CLEC3B acted as a secreting protein that was much reduced in HCC patient, which attracted us to study the role of CLEC3B in HCC.

In order to assess clinical significance of CLEC3B expression in HCC patients, we determined the optimal cutoff value of composite expression score (CES) for IHC staining using receiver operating characteristic (ROC) curve analysis. The optimal cutoff value of CES is 3 (Additional file 2: Figure S2A). CES > 3 referred to high expression of CLEC3B (CLEC3B^high^) and CES < = 3 referred to low expression of CLEC3B (CLEC3B^low^). Down-regulated expression of CLEC3B in HCC patients was significantly related to tumor size, TNM stage, vessel metastasis and depth of invasion, which attested that CLEC3B expression was related to HCC progression, especially to metastasis (Additional file 3: Table S1). Kaplan-Meier analysis of overall survival in 80 HCC patients, revealed that the patients with low expression of CLEC3B showed shorter overall survival time after surgery than patients with high CLEC3B expression, and the median overall survival of CLEC3B^high^ patients was not reached, which is consistent to the analysis for TCGA-LIHC database (Fig. 1f). Moreover, the disease-free survival time after surgery of patients with downregulated CLEC3B expression was significantly shorter than patients with high CLEC3B expression (Additional file 2: Figure S2B), and similar result was observed in the analysis of TCGA-LIHC database (Additional file 2: Figure S2C). The proportion of low expression of CLEC3B was increased from TNM I to TNM IV, and the expression level of CLEC3B was decreased in patients with advanced-stage HCC (Additional file 2: Figure S2D). Meanwhile, CLEC3B is negatively correlated to vessel invasion (Fig. 1g). Taken together, these data indicated that decreased expression of CLEC3B in HCC patients had diagnostic value.

Next, univariate and multivariate analysis was used to estimate the clinical factors which affected overall survival of patients with HCC. As shown in Additional file 4: Table S2 and Additional file 2: Figure S2E, low CLEC3B expression was verified as an independent prognostic factors for overall survival in patients with HCC. Then ROC curve, Harrell’s concordance index (C-index) and Akaike information criterion (AIC) analysis were used to compare the prognostic accuracy of CLEC3B expression and TNM stage alone, or the combination of CLEC3B expression and TNM stage, the results showed the combination of CLEC3B expression and TNM staging system will improve prognostic accuracy and the results of the predictive model for overall survival of HCC patients (Additional file 2: Figure S2F-S2J).

### Down-regulated CLEC3B in HCC-derived exosomes promoted migration, invasion and EMT of HCC cells

**Fig. 2:**
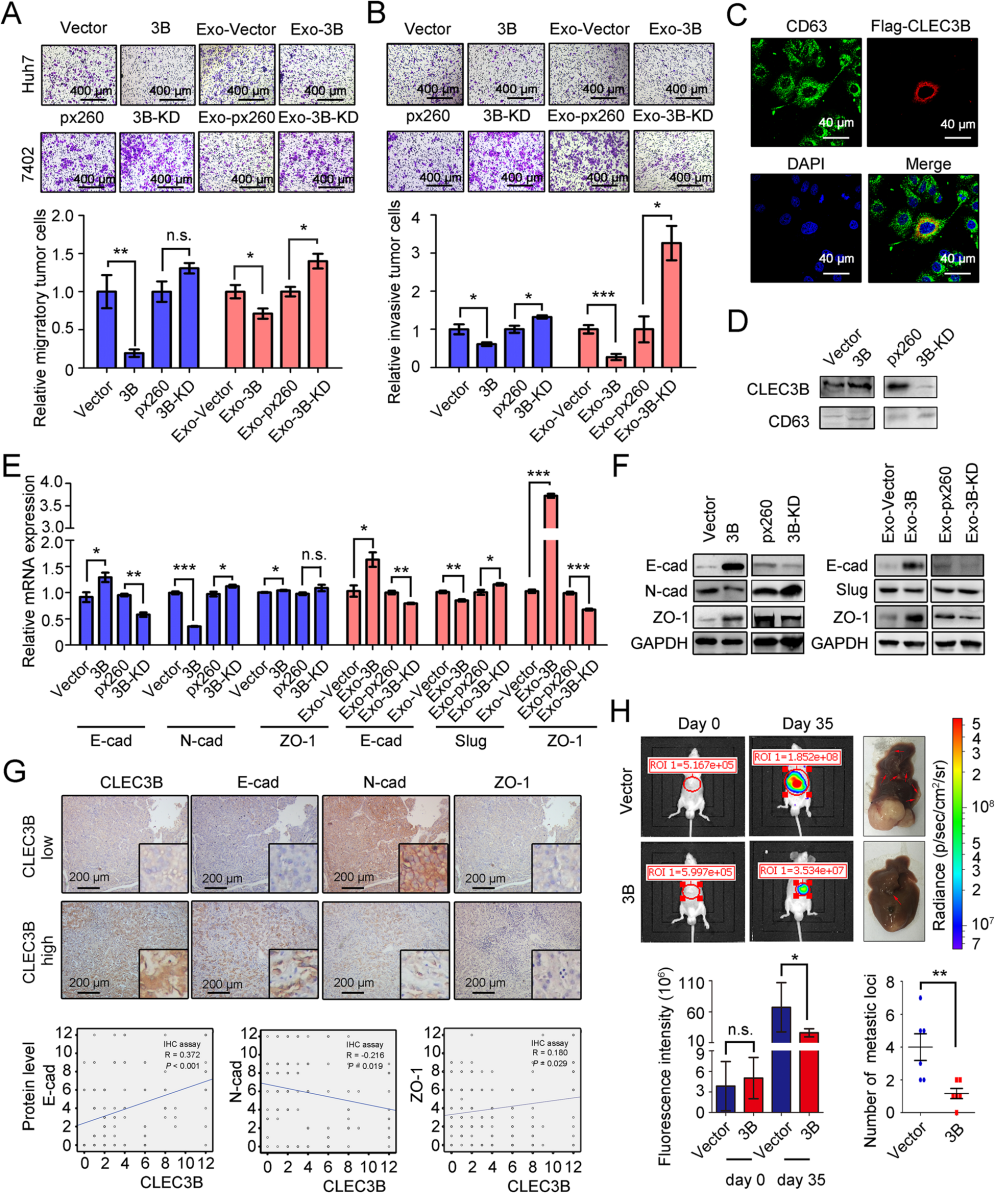
Down-regulated CLEC3B in exosomes promotes migration, invasion and EMT of HCC cells. **a** Representative images and statistical data of relative migratory number of HCC cells with CLEC3B overexpression (Huh-7, 3B, *P* = 0.0086) or CLEC3B knockdown (Bel-7402, 3B-KD, *P* = 0.0542) and treated with exosomes from HCC cells with CLEC3B overexpression (EXO-3B, *P* = 0.0401) or CLEC3B knockdowned (Exo-3B-KD, *P* = 0.0140) in transwell assays. **b** Representative images and statistical data of relative invasive number of HCC cells with 3B (*P* = 0.0185) or 3B-KD (*P* = 0.0198) and treated with EXO-3B (*P* = 0.0003) or Exo-3B-KD (*P* = 0.0140) in invasive assays. **c** Representative images of cellular location of CLEC3B in exosomes. Green, CD63; red, Flag-CLEC3B; blue, DAPI. **d** The protein level of CLEC3B in exosomes secreted by HCC cells. **e** The mRNA expression of E-cad, N-cad and ZO-1 in HCC cells with CLEC3B overexpression (3B; E-cad, *P* = 0.0432; N-cad, *P* < 0.0001; ZO-1, *P* = 0.0485) or knockdown (3B-KD; E-cad, *P* = 0.0017; N-cad, *P* = 0.0461; ZO-1, *P* = 0.1715). The mRNA expression of E-cad (Exo-3B, *P* = 0.0266; Exo-3B-KD, *P* = 0.0047), Slug (Exo-3B, *P* = 0.0069; Exo-3B-KD, *P* = 0.0434) and ZO-1 (Exo-3B, *P* < 0.0001; Exo-3B-KD, *P* = 0.0004) in HCC cells treated with TDEs. **f** The protein expression of EMT relative molecules in HCC cells with CLEC3B overexpression or CLEC3B knockdown and E-cad, Slug and ZO-1 in tumor cells treated with Exo-3B and Exo-3B-KD. **g** Analysis of correlation of CLEC3B with E-cad (R = 0.372, *P* < 0.001), N-cad (R = − 0.216, *P* = 0.019) or ZO-1 (R = 0.180, *P* = 0.029) with IHC staining in hepatocellular carcinoma. **h** In vivo effect of CLEC3B in orthotopic transplantation model. The representative images of tumor-bearing mice (n = 6 each group) were taken at 35 days after tumor transplantation. Statistical data of tumor intensity (day 0, *P* = 0.9237; day 35, *P* = 0.0407) and metastatic loci number (*P* = 0.0088) were shown. 3B, CLEC3B^high^; 3B-KD, CLEC3B^low^; Exo-3B, exosomes from CLEC3B^high^ HCC cells; Exo-3B-KD, exosomes from CLEC3B^low^ HCC cells**.** *. *P* < 0.05; **, *P* < 0.01; ***, *P* < 0.001; n.s., not significant

To analyze effect of CLEC3B in HCC cells we first chose some HCC cell lines to perform experiments. It showed that CLEC3B expressed differently in different HCC cell lines (Additional file 5: Figure S3A and S3B). We detected the efficacy of plasmids in transfected cells at both mRNA level and protein level. Huh-7 cells with low expression of CLEC3B were transfected with CLEC3B overexpressing plasmids, p3xflag-CLEC3B (3B or CLEC3Bhigh), while 7402 cells with high expression were transfected with plasmids, crisper-cas9-px260-CLEC3B, that were used to knockdown expression of CLEC3B in cells (3B-KD or CLEC3Blow) (Additional file 5: Figure S3C and S3D). It was confirmed in IHC staining that CLEC3B was related to HCC metastasis, and wound healing assays and transwell assays were conducted to investigate the migration of HCC cells. We found that migratory cells were inhibited notably in CLEC3Bhigh cells, and increased in CLEC3B knockdown cells in transwell assays (Fig. 2a). In wound healing assays, CLEC3Bhigh cells inhibited migration of HCC cells, while cells with CLEC3Blow showed stronger migratory ability (Additional file 6: Figure S4A). Then, invasion assays were also applied to analyze the invasive ability of HCC cells, and the results showed that invasive cells were decreased in CLEC3Bhigh cells and increased in CLEC3B knockdown cells (Fig. 2b). These results demonstrated that CLEC3B could inhibit migration and invasion of HCC cells.

In view of CLEC3B was a secreted protein [28, 29], thus we collected the supernatants from HCC cells with CLEC3B level changes, and found that the supernatant from CLEC3B^high^ HCC cells (3B) inhibited migration (Additional file 6: Figure S4B and S4C) and invasion of HCC cells (Additional file 6: Figure S4D).

Moreover, similar with previous reports on the location of CLEC3B [38], we also found that CLEC3B co-localized with some CD63-positive secretory vesicles in CLEC3B^high^ Huh7 cells, and the level changes of CLEC3B could be detected in purified exosomes from CLEC3B^high^ (Exo-3B) or CLEC3B^low^ (Exo-3B-KD) cells, which indicated that CLEC3B is a component of exosomes derived from HCC cells (Fig. 2c-d and Additional file 7: Figure S5A). CLEC3B^high^ Exosomes could decrease both migration (Fig. 2a and Additional file 7: Figure S5B) and invasion of HCC cells (Fig. 2).

Since EMT played a vital role in originating migration and invasion of cancer cells, we detected relation between CLEC3B and EMT-associated genes. Firstly, we analyze the relation between CLEC3B level and expression of EMT associating genes in TCGA-LIHC database and IHC staining assays. It showed that both CLEC3B mRNA level and protein level in HCC cells was positive related to E-cadherin (E-cad), ZO-1 and negatively related to N-cadherin (N-cad), that were similar to the results in IHC staining assays (Fig. 2e-g and Additional file 8: Figure S6A-S6C). When HCC cells treated with exosomes derived from HCC cells with CLEC3B^high^ or CLEC3B^low^, it was found that mRNA and protein expression of Slug was negatively correlative to CLEC3B, and E-cad and ZO-1 had positive correlation to CLEC3B (Fig. 2e-f and Additional file 8: Figure S6D-S6E). In vivo data showed that CLEC3B^high^ resulted in decreased intrahepatic metastasis and diminished tumor proliferation (Fig. 2h), which also revealed that CLEC3B suppressed HCC progression. CLEC3B in exosomes can inhibit migratory and invasive ability, EMT of HCC cells via autocrine pathway.

### Exosomal CLEC3B inhibited angiogenesis via reducing VEGF expression of HCC cells

**Fig. 3:**
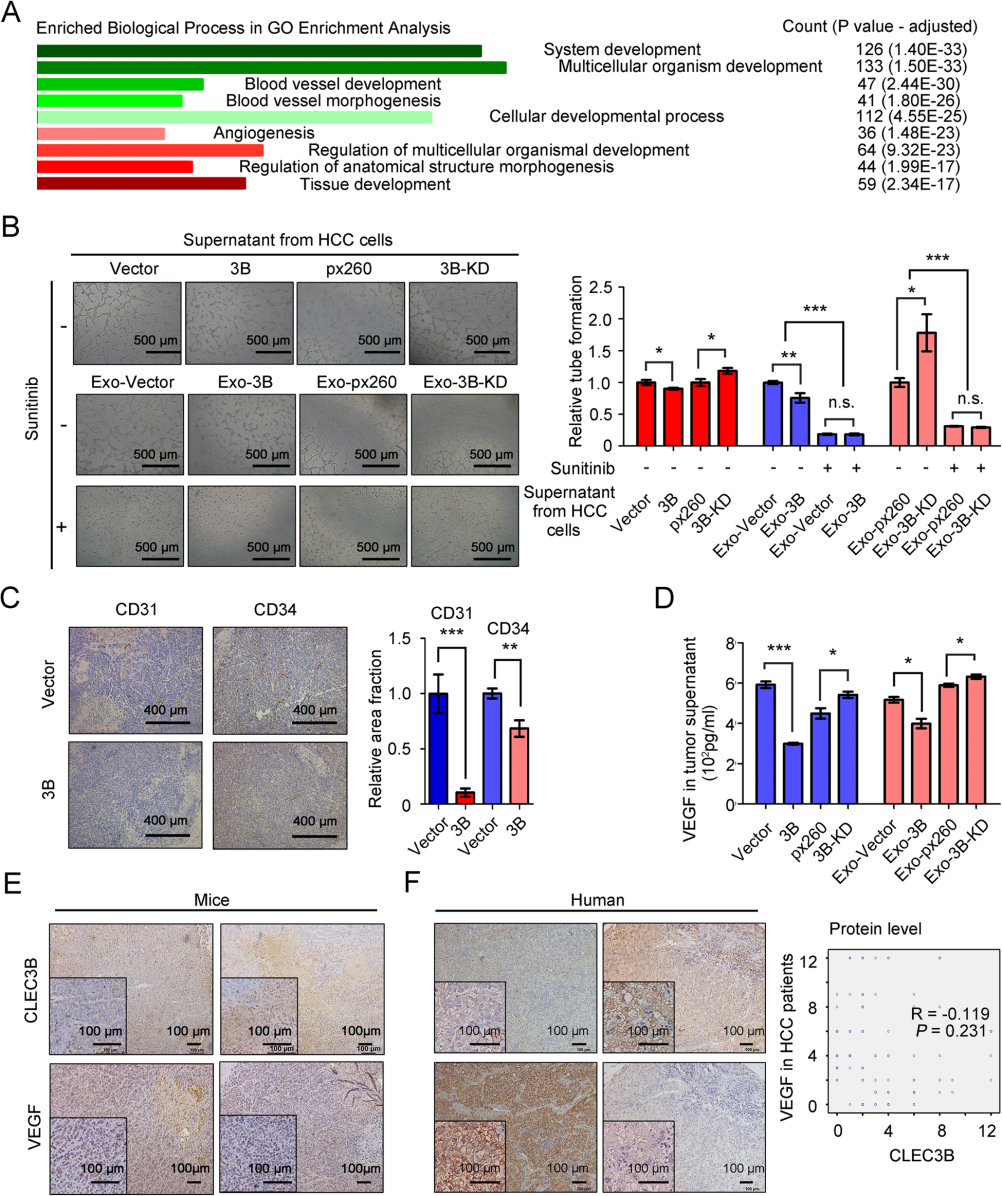
Exosomal CLEC3B decreased VEGF in HCC cells to inhibit angiogenesis. **a** Enriched biological process of CLEC3B-correlated genes in GO Enrichment analysis. **b** The formation of tube-like structures was observed under bright field. Relative tube formation of ECs treated with supernatant from CLEC3B-overexpression (3B, *P* = 0.0265) or CLEC3B-down-regulated (3B-KD, *P* = 0.0225) HCC cells, and Sunitinib and supernatant from Exo-3B treated Huh-7 (no Sunitinib, *P* = 0.0053; Sunitinib, *P* = 0.9313; among groups, *P* < 0.0001) or Exo-3B-KD treated Bel-7402 (no Sunitinib, *P* = 0.0188; Sunitinib, *P* = 0.1518; among groups, *P* = 0.0006). The tube-like structures were determined by pixel density. **c** The representative images and statistic data of CD31 (B, *P* = 0.0005) and CD34 (C, *P* = 0.0065) in HCC of xenografts model. **d** VEGF levels in the supernatant from HCC cells with 3B (*P* < 0.0001) or 3B-KD (*P* = 0.039) and treated with Exo-3B (*P* = 0.0215) or Exo-3B-KD (*P* = 0.0229) determined by ELISA (pg/ml). **e** Representative images and the correlation between CLEC3B and VEGF in mouse liver orthotopic xenografts. **f** Representative images and the correlation between CLEC3B and VEGF (R = − 0.119, *P* = 0.231) in IHC analysis on human HCC tumor samples. *, *P* < 0.05; **, *P* < 0.01; ***, *P* < 0.001; n.s., not significant

To investigate the mechanism how CLEC3B regulated HCC, we further screened out the genes (n = 334) strongly correlated with CLEC3B in TCGA-LIHC database, and performed functional enrichment for these genes (Spearman | *R* | > 0.3 and Pearson | *R* | > 0.3). Enriched biological processes revealed that CLEC3B-correlated genes were closely associated with angiogenesis (36/334) (Fig. 3a, Additional file 9: Figure S7A and Additional file 10: Table S3), which suggested that CLEC3B regulate angiogenesis in HCC. To confirm speculation, we performed tube formation assays using the supernatant from HCC cells, and found that supernatant from CLEC3B^high^ HCC cells induced less tube than that from CLEC3B^Low^ cells (Fig. 3b). IHC assays on the tumor tissues of orthotopic tumor implantation mice also revealed that CD31-positive and CD34-positive ECs significantly decreased in CLEC3B^high^ xenografts (Fig. 3c). CLEC3B^high^ exosomes could significantly restrain angiogenesis in HCC.

VEGF acts as an essential stimulus in tumor angiogenesis, in which VEGF stimulates VEGFR signaling pathways and induces the proliferation and the migration of ECs. The correlation analysis on TCGA dataset revealed that CLEC3B was significantly correlated with VEGF in tumor tissues at mRNA level, and in vitro data also verified that mRNA levels of VEGF were reversely correlative to CLEC3B in HCC cells (Additional file 9: Figure S7B and S7C). However, we failed to detect any cytoplasmic protein level changes of VEGF in WB analysis after CLEC3B overexpression or down-regulated (Additional file 9: Figure S7D). Considering VEGF is a secreting protein, we analyzed the secreting levels of VEGF in tumor supernatants, and found that CLEC3B suppressed the secretion of VEGF (Fig. 3d). Further IHC assays attested that the expression of CLEC3B was negatively correlated with that of VEGF in HCC tumor tissues from patients or tumor implantation mouse (Fig. 3e and f). It indicated that CLEC3B suppress angiogenesis via inhibiting VEGF expression in HCC cells.

It was confirmed that CLEC3B was down-regulated in HCC cells-derived exosomes and CLEC3B would reduce VEGF secretion of HCC cells. We next co-incubated ECs with the supernatants from HCC cells, which were treated with CLEC3B^high^ or CLEC3B^low^ exosomes, and used Sunitinib to block the VEGF-VEGFR signaling. Our data showed that supernatant from HCC cells treated with CLEC3B^low^ exosomes promoted tube formation (Fig. 3b). Moreover, Sunitinib abolished effects of CLEC3B on tube formation (Fig. 3b). Considering that CLEC3B^high^ in HCC cells would significant influence expression of VEGF, then we treated HCC cells with exosomes, and found that supernatant from HCC cells treated with CLEC3B^high^ exosomes obviously reduced mRNA expression and secretion of VEGF in recipient HCC cells, whereas no effects were observed in the cytoplasmic protein levels of VEGF (Additional file 9: Figure S7C-S7D and Fig. 3d). Results revealed that CLEC3B^high^ exosomes might restrain VEGF expression in recipient cells, HCC cells, to reduce angiogenesis.

### Exosomal CLEC3B inhibited metastasis and angiogenesis of ECs

**Fig. 4:**
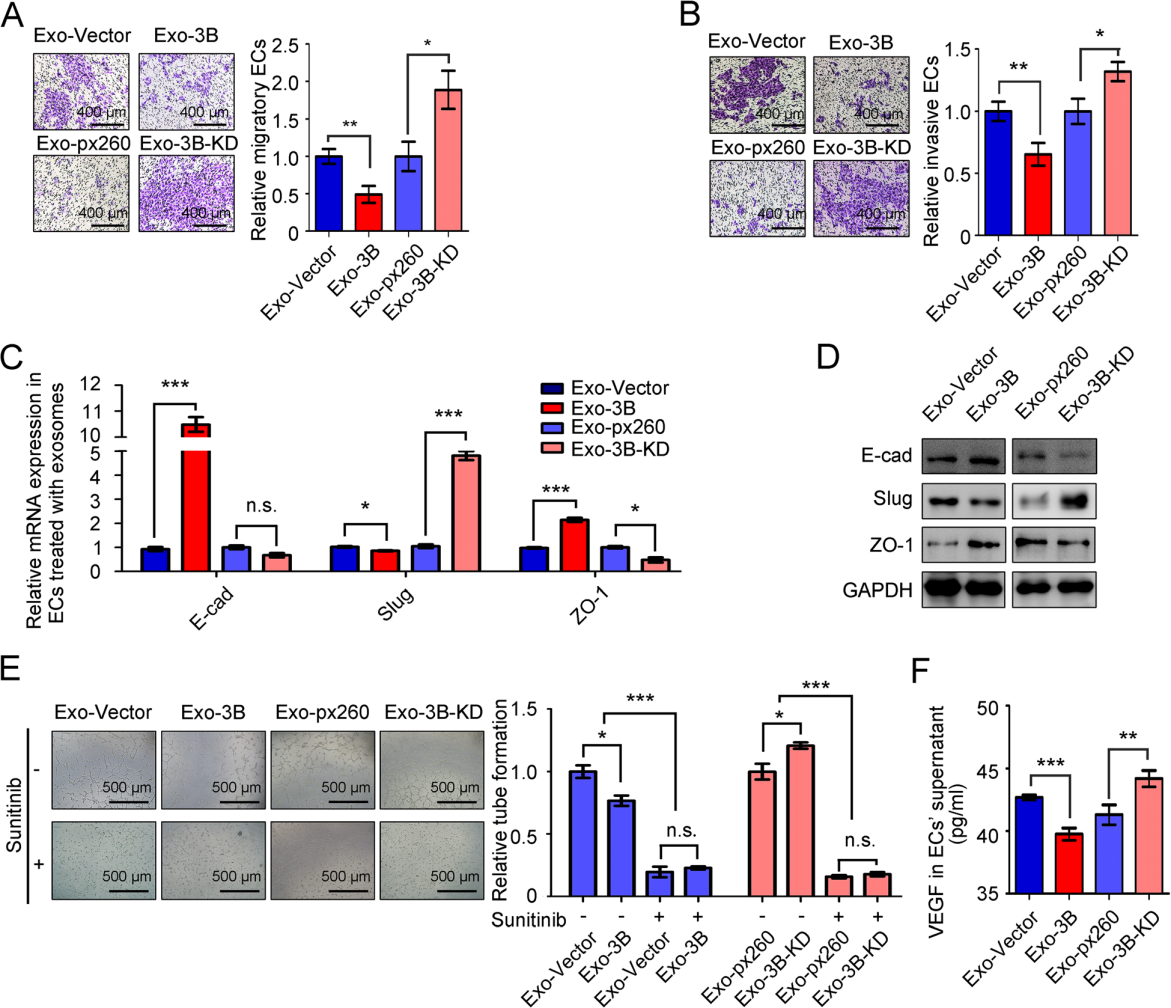
Exosomal CLEC3B suppressed migration, invasion, EMT and angiogenesis of ECs. **a** Representative images and relative migratory number of ECs treated with exosomes from Exo-3B (*P* = 0.0034) or Exo-3B-KD (*P* = 0.0133) in transwell assays. **b** Representative images and relative invasive number of ECs treated with 3B (*P* = 0.0082) or 3B-KD (*P* = 0.0198) in invasive assays. **c** Relative mRNA expression of E-cad (Exo-3B, *P* = 0.0002; Exo-3B-KD, *P* = 0.0497), Slug (Exo-3B, *P* = 0.0159; Exo-3B-KD, *P* < 0.0001) and ZO-1 (Exo-3B, *P* = 0.0002; Exo-3B-KD, *P* = 0.0110) in ECs treated with exosomes. **d** The protein level of E-cad, Slug and ZO-1 in ECs treated with exosomes. **e** Tube formation of ECs treated with Sunitinib, Exo-3B (no Sunitinib, *P* = 0.0238; Sunitinib, *P* = 0.4988; among groups, *P* < 0.0001) or Exo-3B-KD (no Sunitinib, *P* = 0.0191; Sunitinib, *P* = 0.4711; among groups, *P* < 0.0001). **f** VEGF in supernatants of ECs treated with exosomes determined by ELISA (Exo-3B, *P* = 0.0002; Exo-3B-KD, *P* = 0.0011). *, *P* < 0.05; **, *P* < 0.01; ***, *P* < 0.001; n.s., not significant

In view of the effect of CLEC3B on HCC cells previously, ECs were cultured with supernatant from HCC cells and it showed that supernatant from CLEC3B^high^ HCC cells significantly inhibited migratory and invasive ability of ECs (Additional file 11: Figure S8A-S8B). It was reported that VEGF secreted by tumor cells also facilitated an EMT phenotype recipient cells [10], and then we isolated exosomes from HCC supernatant and directly co-cultured with ECs. It was found that CLEC3B^high^ exosomes inhibited migration and invasion of ECs (Additional file 11: Figure S8C and Fig. 4a-b).

As mentioned above, exosomal CLEC3B elevated the expression of E-cad, ZO-1 and decreased Slug, and eventually weaken the migratory and invasive ability of HCC cells (Fig. 2d-e). In ECs, we detected that CLEC3B had positive correlation to E-cad and ZO-1, while negatively correlative to Slug (Fig. 4c-d and Additional file 11: Figure S8D-S8E), verifying that exosomal CLEC3B directly inhibits migration and invasion of ECs. Exosomal CLEC3B reversely regulated EMT in ECs.

Tube formation assays revealed that exosomal CLEC3B also inhibit angiogenesis when directly treat ECs (Fig. 4e). Similar with the effect on tumor cells, exosomal CLEC3B down-regulated mRNA expression and secretion of VEGF in ECs, and the cytoplasmic protein level of VEGF in ECs was decreased by exosomal CLEC3B either (Additional file 11: Figure S8F-S8G and Fig. 4f). Moreover, Sunitinib treatment was able to block the effects of exosomal CLEC3B on tube formation of ECs (Fig. 4e), attesting that exosomal CLEC3B could directly target on ECs and reduced secretion of VEGF of ECs to inhibit angiogenesis.

### Exosomal CLEC3B reduced VEGF via activating AMPK signaling in HCC cells

**Fig. 5:**
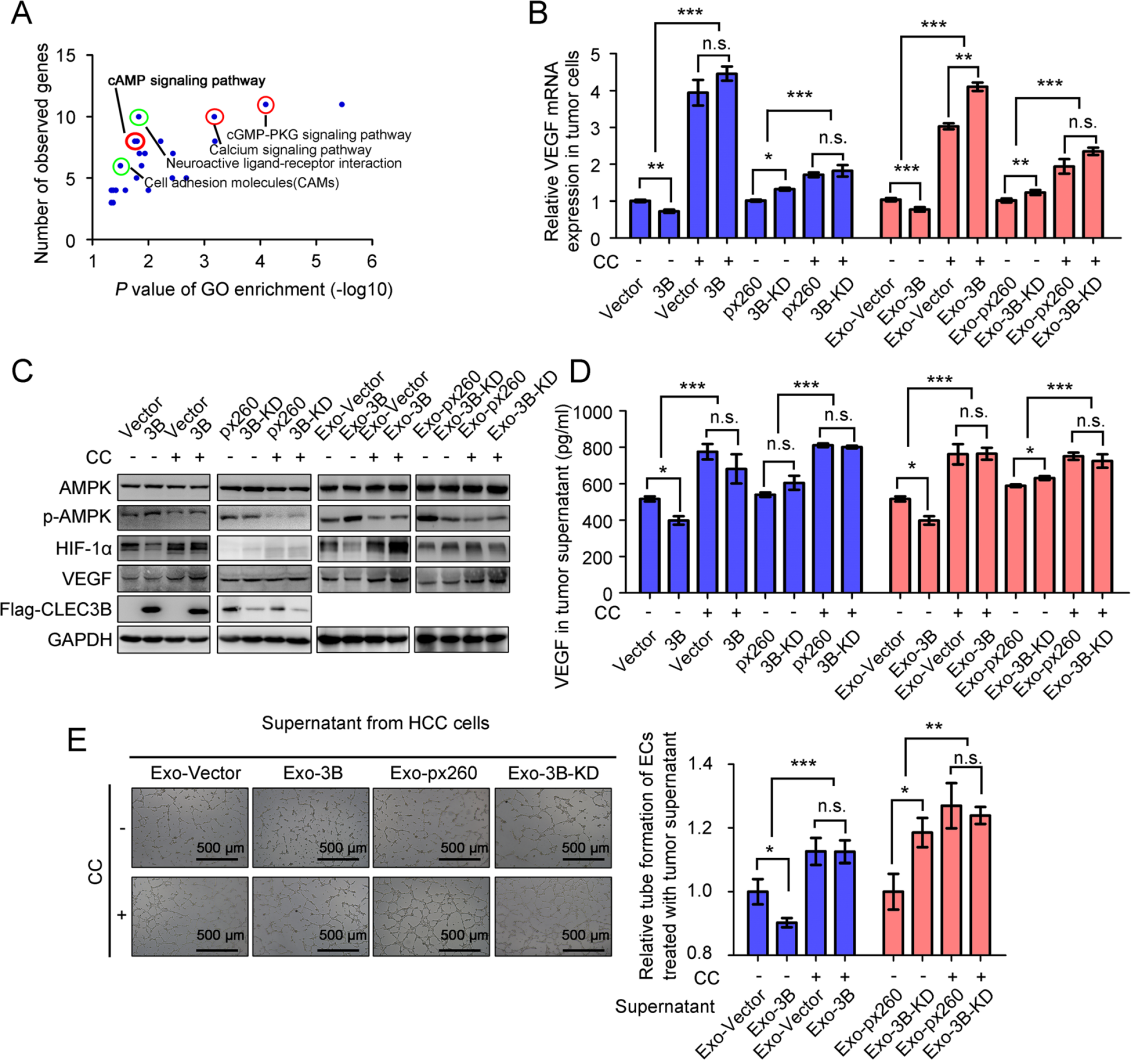
Exosomal CLEC3B decreased VEGF via activating phosphorylation of AMPK in HCC cells. **a** KEGG enrichment of CLEC3B-correlated genes in TCGA-LIHC database. Points with circled marks were related to Environmental Information Processing, and red related to Signaling transduction while green related to Signaling molecules and interaction. **b** Relative mRNA expression of VEGF in tumor cells with 3B (no CC, *P* = 0.0022; CC, *P* = 0.2697; among groups, *P* < 0.0001) or 3B-KD (no CC, *P* = 0.0372; CC, *P* = 0.1357; among groups, *P* = 0.0001) and tumor cells treated with Exo-3B (CC, *P* = 0.0005; no CC, *P* = 0.0016; among groups, *P* < 0.0001) or Exo-3B-KD (CC, *P* = 0.009; no CC, *P* = 0.0784; among groups, *P* < 0.0001). **c** Protein levels in HCC cells with 3B or 3B-KD and treated with Exo-3B or Exo-3B-KD, Compound C (CC) or not. **d** Secretion of VEGF in supernatants of tumor cells with 3B (no CC, *P* = 0.0101; CC, *P* = 0.3572; among groups, *P* = 0.0004) or 3B-KD (no CC, *P* = 0.1832; CC, *P* = 0.4912; among groups, *P* < 0.0001) and treated with Exo-3B (no CC, *P* = 0.0123; CC, *P* = 0.1702; among groups, *P* < 0.0001) or Exo-3B-KD (no CC, *P* = 0.0340; CC, *P* = 0.5750; among groups, *P* = 0.0004). **e** Relative tube formation of ECs treated with supernatant from recipient Huh7 cells treated with Exo-3B (no CC, *P* = 0.0265; CC, *P* = 0.9874; among groups, *P* < 0.0001) or Exo-3B-KD (no CC, *P* = 0.0281; CC, *P* = 0.6746; among groups, *P* = 0.0045). *, *P* < 0.05; **, *P* < 0.01; ***, *P* < 0.001; n.s., not significant

When analyzing the functional enrichments of CLEC3B-correlated genes in TCGA-LIHC database, we found that CLEC3B was participated in some signaling pathways, such as calcium signaling pathway, cGMP-PKG signaling pathway and cAMP signaling pathway. It was showed that elevated cAMP was able to activate AMPK signaling pathway [18] (Fig. 5a, Additional file 10: Table S3, Additional file 12: Figure S9A and Additional file 13: Table S4). In vitro data revealed that phosphorylated AMPK (p-AMPK) was increased in CLEC3B^high^ HCC cells (Additional file 12: Figure S9B), which also indicated AMPK pathway is regulated by CLEC3B. Phosphorylation of Thr172 in AMPKα is required in activation of AMPK [39]. To further determine whether AMPK pathway is involved in the biological functions of CLEC3B in HCC, We used Compound C (CC), an inhibitor suppressed activation of AMPK, to treat HCC cells. Results showed that CC significantly influenced the effects of overexpressed or down-regulated CLEC3B on regulating expression of AMPK downstream effectors, like HIF1α, and ultimately VEGF synthesis and secretion of HCC cells (Fig. 5b-d). Effects of exosomal CLEC3B^high^ on VEGF synthesis and secretion in HCC cells could also be diminished when AMPK was inhibited (Fig. 5b-d). Tube formation assays using the supernatants from exosome-treated HCC cells showed that CC attenuated the effects of CLEC3B either (Fig. 5e).

**Fig. 6:**
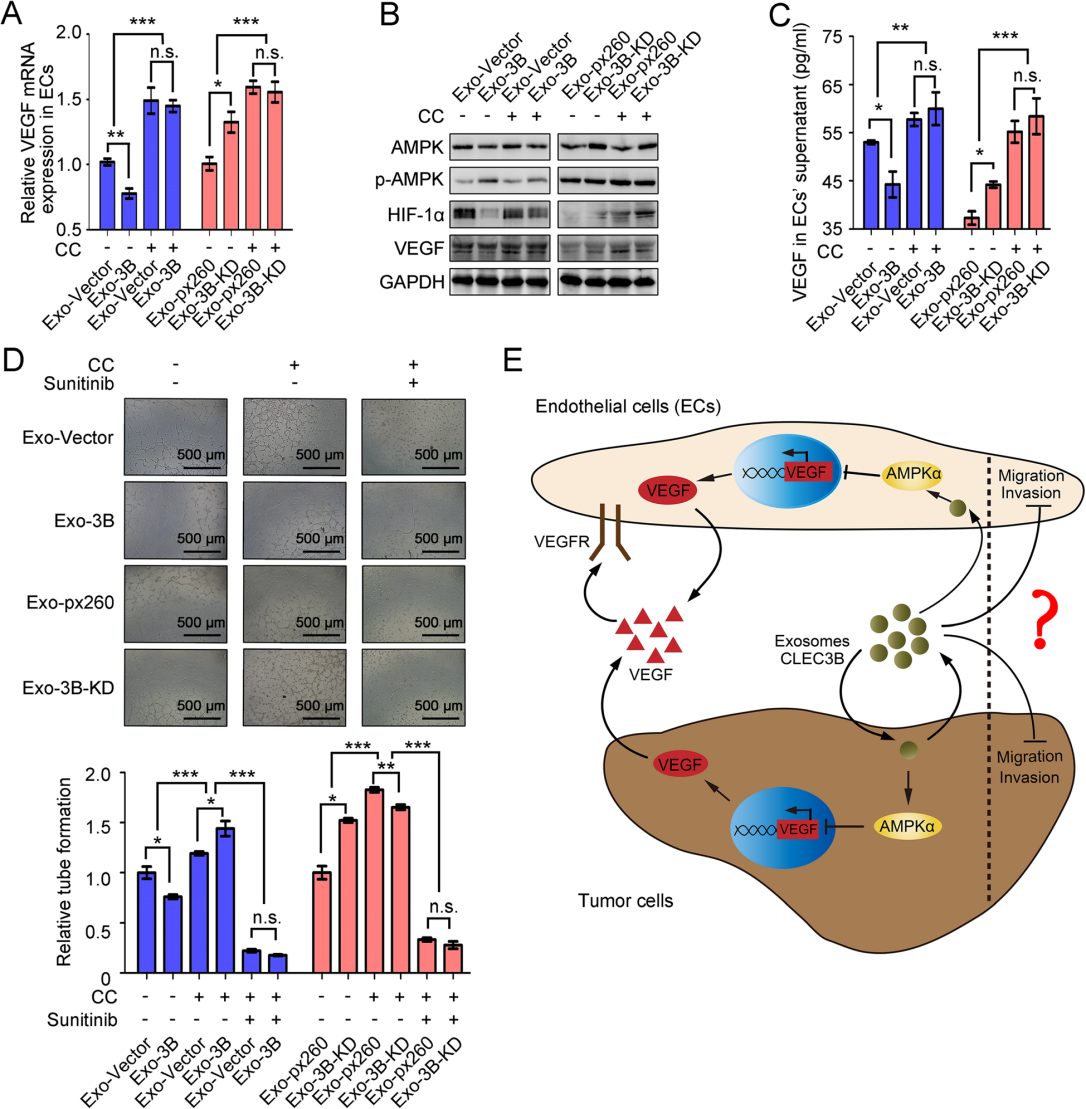
Exosomal CLEC3B decreased VEGF via activating phosphorylation of AMPK in ECs. **a** mRNA expression of VEGF in ECs treated with Exo-3B (no CC, *P* = 0.0065; CC, *P* = 0.7226; among groups, *P* < 0.0001) or Exo-3B-KD (no CC, *P* = 0.0063; CC, *P* = 0.7087; among groups, *P* < 0.0001). **b** Protein level changes in ECs treated with CC or not and Exo-3B or Exo-3B-KD, which were isolated from tumor cells. **c** The secretion of VEGF from ECs treated with Exo-3B (no CC, *P* = 0.0320; CC, *P* = 0.5765; among groups, *P* = 0.0021) or Exo-3B-KD (no CC, *P* = 0.0105; CC, *P* = 0.5004; among groups, *P* < 0.0001). **d** Tube formation of ECs treated with CC, Sunitinib and Exo-3B (no CC, no Sunitinib, *P* = 0.0204; CC, no Sunitinib, *P* = 0.0240; CC, Sunitinib, *P* = 0.0573; group no CC and CC, *P* < 0.0001; group no Sunitinib and Sunitinib, *P* < 0.0001) or Exo-3B-KD (no CC, no Sunitinib, *P* = 0.0017; CC, no Sunitinib, *P* = 0.0087; CC, Sunitinib, *P* = 0.3094; group no CC and CC, *P* < 0.0001; group no Sunitinib and Sunitinib, *P* < 0.0001). **e** Schematic graph for the functions of CLEC3B in HCC. *, *P* < 0.05; **, *P* < 0.01; ***, *P* < 0.001; n.s., not significant

In addition to the influences on tumor cells, we also observed that inhibition of p-AMPK lessened exosomal CLEC3B-mediated regulation of VEGF synthesis and secretion in ECs, as well as tube formation when directly treating ECs with exosomes from HCC cells (Fig. 6a-d). These data indicated that exosomal CLEC3B regulates VEGF synthesis through AMPK pathway in target cells, both HCC cells and ECs.

Intriguingly, although exosomal CLEC3B down-regulated the migration and invasion of HCC cells and ECs in a similar manner, inhibition of p-AMPK conferred no significant effect on CLEC3B-mediated EMT, migration or invasion in HCC cells (Additional file 12: Figure S9C-S9F), indicating that there might be some other pathways critical for the effects of exosomal CLEC3B on EMT. Similarly, exosomal CLEC3B inhibiting migratory, invasive ability of ECs was independent of AMPK signaling, as well as EMT (Additional file 14: Figure S10A-S10D).

## Discussion

Exosomes are critical mediators of signals in tumor initiation and development, and can be used to predict and treat cancer [40–43]. However, research on diagnostic and prognostic exosome biomarkers of HCC is still at the initial phase. In this study, we determined CLEC3B as an exosome protein in HCC, and attested that exosomal CLEC3B^high^ inhibits migration and invasion via regulating EMT process in both HCC cells and ECs, and inhibits tube formation of ECs via downregulating VEGF from exosomes-recipient cells. Our findings demonstrated that exosomal CLEC3B^high^ was a tumor suppressor, especially in targeting VEGF pathway in HCC.

VEGF-targeting strategies in cancer treatment is well-developed in recent years. Inhibitors to VEGF not only stop angiogenesis, but also return some tumor vessels to normal and Sunitinib has been recently approved by the US Food and Drug Administration (FDA) for clinical use to anti-VEGF [44]. However, there are still some disadvantages when applying Sunitinib including its side effects and drug resistance. Developing complementary medicine for VEGF-targeting therapy in urgently needed. Exosome could deliver medicine to tumor efficiently and accurately. Despite the promoting effects of TDEs on angiogenesis, since besides tumor cells, hepatocytes, nonparenchymal liver cells and immune cells also secret exosomes in HCC, purifying the CLEC3B^high^ exosomes from these non-tumor cells and applying these exosome in HCC treatments, especially in VEGF-targeting therapy, might have promising prospect [16, 45–47].

Here, AMPK was proved as one of the common downstream signals of exosomal CLEC3B in both tumor cells and ECs. AMPK can be activated to limit cell proliferation upon energy stress induced by glucose depletion [48]. AMPK is a important therapeutic target for metabolic syndrome and type-2 diabetes, and it is suggested that AMPK is a potential tumor suppressor [49]. Glucose-AMPK-TET2-5hmC axis participate in anti-tumor effect of metformin, which is a first-line antidiabetic drug. AMPK regulates proliferation, EMT and apoptosis through AMPK/mTOR, AMPK/PKA/GSK-3β or other signaling pathway [23, 50]. It showed that exosomal CLEC3B enhances activation of AMPK, demonstrating the suppressive roles of CLEC3B in HCC and CLEC3B^high^ exosomes might be a potential therapy for cancers, even diabetes. Although exosomal CLEC3B activates AMPK and inhibits EMT in different cells at the same time, AMPK inhibitor fails to inhibit EMT, migration and invasion, indicated there might be other pathway involved in exosomal CLEC3B.

Although our results indicated that CLEC3B^high^ exosomes suppressed metastasis of target cells and angiogenesis, and suggested that CLEC3B is involved in the signal transduction during exosome-ECs interaction, we fail to sufficient evidences to prove whether functions of exosomes directly depend on exosomal CLEC3B. Actually, in HCC, both RNA and protein within exosomes are different between HCC cells and untransformed cells [14]. CLEC3B^low^ in HCC cells might change expression of miRNA, lncRNA, circRNA and various protein before packaged into exosomes. CLEC3B acts as an exosomal protein may alter other biomolecules sort and secret into exosomes, and even modulates selective endocytosis and membrane fusion of exosomes with target cells. Contradictorily, a recent study reported that secreted CLEC3B from cancer-associated fibroblast promoted the migration of tumor cells in colorectal cancer [51]. The contradictory effects of CLEC3B might result from the specific inclusions in CLEC3B-positive exosomes from different donated cells. Further studies are required to determine the detailed downstream of exosomal CLEC3B.

## Conclusions

In conclusion, this study demonstrated that CLEC3B, which was down-regulated in HCC, acted as a tumor marker for poor prognostic factor for HCC patients and played a significant role in target cells and microenvironmental remodeling in HCC via exosomes. Mechanically, exosomal CLEC3B promoted the phosphorylation of AMPK, which decreased expression of VEGF in both HCC cells and ECs, and eventually alleviated angiogenesis. Meanwhile, exosomal CLEC3B could mitigate migration and invasion of recipient cells, but function of exosomal CLEC3B on inhibiting metastasis of target cells need to be further determined in vivo.

## Additional files

Additional file 1: **Figure S1.** CLEC3B was down-regulated in serum of HCC patients. (A) Relative quantity of CLEC3B in plasma of the normal (N) and HCC (T) patients. Western Blot was used to determine CLEC3B while total preotein was performed with Ponceau S staining (*P* = 0.0425). (TIF 2215 kb)

Additional file 2: **Figure S2.** Correlation between CLEC3B expression and clinicopathological characteristic, and improvement of the TNM staging prognostic model with CLEC3B expression. (A) Receiver operating characteristic (ROC) curve analyses of different cutoff values of composite expression score (CES), and the area under the ROC curve (AUC), 95% confident interval (95% CI) and *P*-value are shown. (B, C) The disease free time in IHC staining (n = 80, *P* < 0.0001) (B) and TCGA-LIHC database (n = 315, *P* < 0.0001) (C), based on CLEC3B expression level, were calculated by Kaplan–Meier. (D) The relative proportion of patients with low CLEC3B expression is increased with the tumor progression in hepatocellular carcinoma (*P* = 0.006). (E) Multivariate Cox analysis was conducted to analyze independent prognostic factors in patients with hepatocellular carcinoma. (F) ROC analysis of the sensitivity and specificity for the predictive value of CLEC3B expression model, TNM model and the combined model of CLEC3B and TNM. (G) AIC and C-index, another prognostic predicting model nomogram for overall survival, were performed to analyze the predictive accuracies of TNM stage, CLEC3B expression and the combined model of CLEC3B and TNM. (H) Nomogram was built to quantify the combined effect of the proven independent prognostic factors for overall survival. (I) Calibration plot of the nomogram for 5-year survival. (J) Of all patients, three groups were divided according to the total points in the nomogram which range of 0–40, 41–120, 121–160, was refined as low risk, medium and high risk subgroup (*P* < 0.0001). Kaplan–Meier analysis was used to test the correlation of the risk with overall survival. *, *P* < 0.05; **, *P* < 0.01; ***, *P* < 0.001; n.s., not significant. (TIF 827 kb)

Additional file 3: **Table S1.** CLEC3B expression and relative factors. (DOCX 15 kb)

Additional file 4: **Table S2.** Survival time of HCC patients and relative factors. (DOCX 16 kb)

Additional file 5: **Figure S3.** CLEC3B was significantly decreased in HCC cells. (A, B) The mRNA and protein level of CLEC3B in different hepatocellular carcinoma cell lines were analyzed by real-time polymerase chain reaction (RT-PCR) (A) and western blot (B). (C) The overexpression (*P* = 0.0005) and knockdown (*P* = 0.0018) efficiency of relative mRNA expression of plasmids in HCC cells. (D) The overexpression and knockdown efficiency of protein expression of plasmids in HCC cells. *, *P* < 0.05; **, *P* < 0.01; ***, *P* < 0.001; n.s., not significant. (TIF 657 kb)

Additional file 6: **Figure S4.** Down-regulated CLEC3B in HCC promoted migration and invasion of HCC cells via supernatant. (A) Representative images and statistical data of migratory distance of HCC cells with CLEC3B overexpression (3B, *P* < 0.0001) or CLEC3B knockdown (3B-KD, *P* < 0.0001) using wound healing assay. (B) Representative images and migratory distance of HCC cells treated with the supernatant from HCC cells with CLEC3B overexpression (3B; *P* = 0.0603) or down-regulated (3B-KD; *P* = 0.0572) in wound healing assays. (C) Representative images and migratory number of HCC cells treated with supernatant from 3B (*P* = 0.009) or 3B-KD (*P* = 0.0087) tumor cells using transwell assays. (D) Representative images and invasive number of HCC cells treated with supernatant from 3B (*P* = 0.015) or 3B-KD (*P* = 0.3806) tumor cells using invasive assays. *, *P* < 0.05; **, *P* < 0.01; ***, *P* < 0.001; n.s., not significant. (TIF 4362 kb)

Additional file 7: **Figure S5.** CLEC3B was significantly down-regulated in exosomes derived from HCC. (A) Representative micrograph of exosomes derived from 3B cells. (B) Representative images and statistical data of migratory distance of HCC cells treated with exosomes from HCC cells with 3B (Exo-3B, *P* = 0.0001) or 3B-KD (Exo-3B-KD, *P* = 0.0079) using wound healing assay. *, *P* < 0.05; **, *P* < 0.01; ***, *P* < 0.001; n.s., not significant. (TIF 1851 kb)

Additional file 8: **Figure S6.** Exosomal CLEC3B inhibited EMT of HCC cells. (A) Analysis of correlation of CLEC3B with E-cad (R = 0.107, *P* = 0.04), ZO-1 (R = 0.002, *P* = 0.972), N-cad (R = − 0.116, *P* = 0.026), Snai1 (R = 0.438, *P* < 0.001), Slug (R = 0.147, *P* = 0.005),β-catenin (R = − 0.101, *P* = 0.051) and Vimentin (R = 0.401, *P* < 0.001) in TCGA-LIHC database. (B) The relative mRNA expression of EMT relative molecules in HCC cells transfected with 3B (Snai1, *P* = 0.0010; Slug, *P* = 0.0002; Vimentin, *P* = 0.0107; β-catenin, *P* = 0.0023) or 3B-KD (Snai1, *P* = 0.7509; Slug, *P* = 0.0100; Vimentin, *P* = 0.6157; β-catenin, *P* = 0.7604) plasmids. (C) The protein expression of EMT relative molecules in HCC cells transfected with 3B or 3B-KD plasmids. (D) The mRNA expression of N-cad (Exo-3B, *P* < 0.0001; Exo-3B-KD, *P* = 0.0015), Snai1 (Exo-3B, *P* = 0.0011; Exo-3B-KD, *P* = 0.0010), β-catenin (Exo-3B, *P* = 0.0015; Exo-3B-KD, *P* = 0.1158) and Vimentin (Exo-3B, *P* = 0.1211; Exo-3B-KD, *P* = 0.7113) in tumor cells, which were treated with exosomes. (E) Levels of protein related to EMT in tumor cells treated with Exo-3B or Exo-3B-KD. *, *P* < 0.05; **, *P* < 0.01; ***, *P* < 0.001; n.s., not significant. (TIF 1974 kb)

Additional file 9: **Figure S7.** Exosomal CLEC3B decreased VEGF in HCC cells to inhibit angiogenesis. (A) Enriched biological process of genes significantly correlated with CLEC3B in TCGA database. (B) Correlation between CLEC3B and VEGF (Pearson, R = − 0.475, *P* < 0.001) in TCGA-LIHC database. (C) Relative mRNA expression of VEGF in HCC cells with 3B (*P* < 0.001) or 3B-KD (*P* = 0.0183), and treated with Exo-3B (*P* < 0.0001) or 3B-KD (*P* = 0.0009). (D) Protein levels of VEGF in 3B or 3B-KD HCC cells or HCC cells treated with Exo-3B-treated or Exo-3B-KD. *, *P* < 0.05; **, *P* < 0.01; ***, *P* < 0.001; n.s, not significant. (TIF 973 kb)

Additional file 10: **Table S3.** Genes correlated to CLEC3B in TCGA database. (XLSX 1010 kb)

Additional file 11: **Figure S8.** Exosomal CLEC3B inhibited migration, invasion and EMT of ECs. (A) Representative images and relative migratory number of ECs treated with supernatant from 3B (*P* = 0.0003) or 3B-KD (*P* = 0.0191) HCC tumor cells in transwell assays. (B) Representative images and relative invasive number of ECs treated with supernatant from 3B (*P* = 0.0029) or 3B-KD (*P* = 0.0011) tumor cells in invasive assays. (C) Representative images and relative migratory distance of ECs treated with Exo-3B (*P* = 0.0033) or Exo-3B-KD (*P* = 0.0377) in wound healing assays. (D) The relative mRNA expression of N-cad (Exo-3B, *P* = 0.0287; Exo-3B-KD, *P* = 0.0014), Snai1 (Exo-3B, *P* = 0.0004; Exo-3B-KD, *P* = 0.0014), β-catenin (Exo-3B, *P* < 0.0001; Exo-3B-KD, *P* = 0.0166) and Vimentin (Exo-3B, *P* = 0.0003; Exo-3B-KD, *P* = 0.0139) in ECs treated with exosomes from tumor cells. (E) The protein level of N-cad, Snai1, β-catenin and Vimentin in ECs treated with Exo-3B or Exo-3B-KD from tumor cells. (F) Relative VEGF mRNA expression in ECs treated with exosomes (Exo-3B, *P* = 0.0075; Exo-3B-KD, *P* = 0.0001). (G) Protein level of VEGF in ECs treated with exosomes. *, *P* < 0.05; **, *P* < 0.01; ***, *P* < 0.001; n.s., not significant. (TIF 3752 kb)

Additional file 12: **Figure S9.** Exosomal CLEC3B inhibited migration, invasion and EMT independent of AMPK signaling pathway in HCC cells. (A) KEGG enrichment CLEC3B-correlated genes. A: Metabolism; AF: metabolism of other amino acids; AK: Xenobiotics biodegradation and metabolism; C: Environmental Information Processing; CB: Signaling transduction; CC: Signaling molecules and interaction; D: Cellular Processes; DD: Cellular community; E: Organismal Systems; EA: Immune system; EB: Endocrine system; EC: Circulatory system; EF: Nervous system; EG: Sensory system; F: Human Diseases; FD: Neurodegenerative diseases; FE: Substance dependence; FG: Endocrine and metabolic diseases; H: Other and unknown; HA: Other and unknown. (B) Expression of proteins in tumor cells affected by CLEC3B. (C) Relative mRNA expression of E-cad (Exo-3B, no CC, *P* = 0.0266; CC, *P* = 0.0491; Exo-3B-KD, no CC, *P* = 0.0473; CC, *P* = 0.1337), Slug (Exo-3B, *P* = 0.0069, *P* = 0.0019; Exo-3B-KD, *P* = 0.0040, *P* = 0.0016) and ZO-1 (Exo-3B, *P* < 0.0001, *P* = 0.0016; Exo-3B-KD, *P* = 0.0008, *P* = 0.9323) in tumor cells treated with Compound C (CC, a drug to inhibit phosphorylation of AMPK) and EXO-3B or Exo-3B-KD. (D) Protein expression of E-cad, Slug and ZO-1 in tumor cells treated with CC and EXO-3B or Exo-3B-KD. (E) Representative images and relative migratory number of tumor cells treated with CC or not and Exo-3B (no CC, *P* = 0.0401; CC, *P* = 0.0165) or Exo-3B-KD (no CC, *P* = 0.0140; CC *P* = 0.0024). (F) Representative images and relative invasive number of tumor cells treated with CC or not and Exo-3B (no CC, *P* = 0.0003; CC, *P* = 0.0618) or Exo-3B-KD (no CC, *P* = 0.0409; CC, *P* = 0.0005). *, *P* < 0.05; **, *P* < 0.01; ***, *P* < 0.001; n.s., not significant. (TIF 5213 kb)

Additional file 13: **Table S4.** The functional enrichment of KEGG pathway. (XLSX 27 kb)

Additional file 14: **Figure S10.** Exosomal CLEC3B inhibiting migration, invasion and EMT were AMPK signaling-independent in ECs. (A) Representative images and relative migratory number of ECs incubated with CC and exosomes from tumor cells, Exo-3B (no CC, *P* = 0.0003; CC, *P* = 0.3142) and Exo-3B-KD (no CC, *P* = 0.0389; CC, *P* = 0.4269). (B) Representative images and relative invasive number of ECs incubated with CC and Exo-3B (no CC, *P* = 0.0029; CC, *P* = 0.2830) or Exo-3B-KD (no CC, *P* = 0.0011; CC, *P* = 0.0733). (C) Relative mRNA expression of E-cad (Exo-3B, no CC, *P* = 0.0002; CC, *P* = 0.4442; Exo-3B-KD, no CC, *P* = 0.0509; CC, *P* = 0.0002), Slug (Exo-3B, *P* = 0.0159, *P* = 0.0030; Exo-3B-KD, no CC, *P* < 0.0001; CC, *P* = 0.0920) and ZO-1 (Exo-3B, no CC, *P* = 0.0002; CC, *P* < 0.0001; Exo-3B-KD, no CC, *P* = 0.0110; CC, *P* = 0.0134) in ECs treated with CC and Exo-3B or Exo-3B-KD. (D) Expression of E-cad, Slug and ZO-1 in ECs treated with CC and Exo-3B or Exo-3B-KD. *, *P* < 0.05; **, *P* < 0.01; ***, *P* < 0.001; n.s., not significant. (TIF 4765 kb)

## Data Availability

Materials described in the manuscript will be freely available to any scientist wishing to use them for non-commercial purposes, without breaching participant confidentiality.

## Abbreviations

AMPK: AMP-activated Protein Kinase
CC: Compound C
CI: Confidence Interval
CLEC3B: C-Type Lectin Domain Family 3 Member B
E-cad: E-cadherin
ECs: Endothelial cells
EMT: Epithelial-mesenchymal transition
Exo: Exosomes
GAPGH: Glyceraldehyde-3-phosphate dehydrogenase
GEO: Gene Expression Omnibus
HCC: Hepatocellular carcinoma
HIF-1: Hypoxia inducible factor-1
HR: Hazard Ratio
N-cad: N-cadherin
TCGA-LIHC: The Cancer Genome Altas-liver hepatocellular carcinoma
TDEs: Tumor-derived exosomes
VEGF: Vascular endothelial cell growth factor
VEGFR: Vascular Endothelial cell growth factor receptor
