# Supplementary figures and images for: Downregulation of exosomal CLEC3B in hepatocellular carcinoma promotes metastasis and angiogenesis via AMPK and VEGF signals
Source: Cell Commun Signal. 2019 Sep 2;17:113. doi: 10.1186/s12964-019-0423-6 (PMC6721425; doi:10.1186/s12964-019-0423-6)

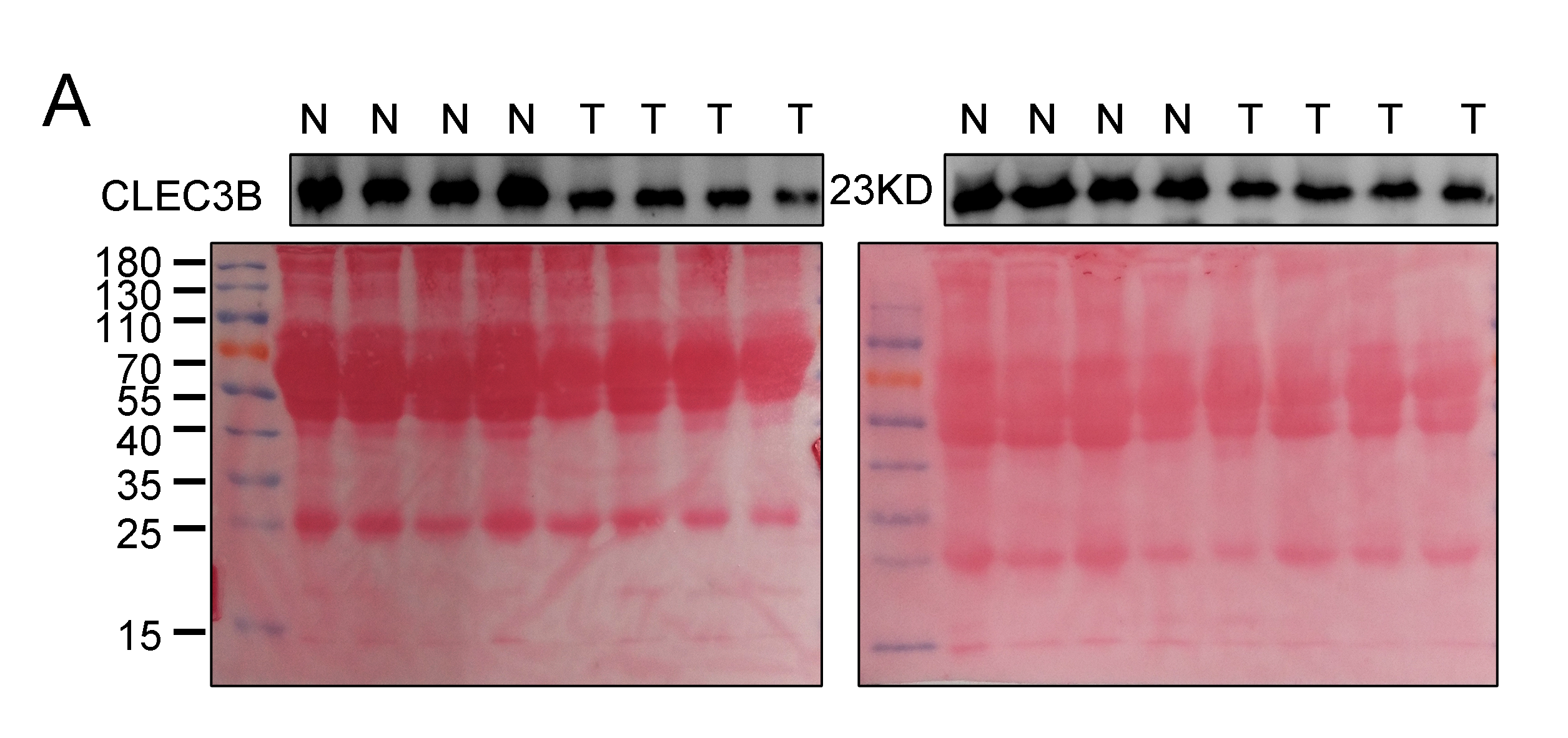

Supplement: Supplementary file 1 — Figure S1. CLEC3B was down-regulated in serum of HCC patients. (A) Relative quantity of CLEC3B in plasma of the normal (N) and HCC (T) patients. Western Blot was used to determine CLEC3B while total preotein was performed with Ponceau S staining (P = 0.0425). (TIF 2215 kb) [file 12964_2019_423_MOESM1_ESM.tif]

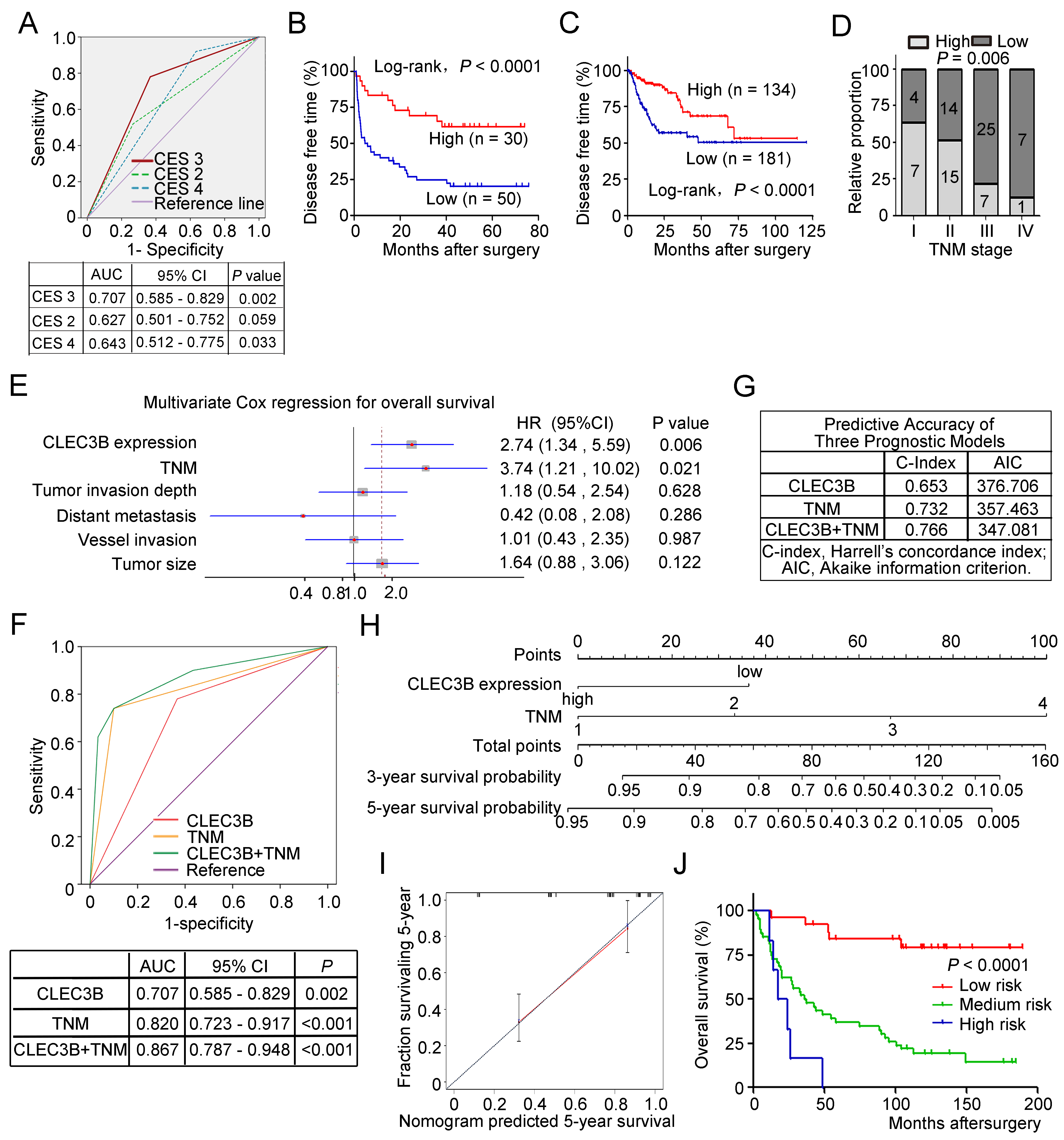

Supplement: Supplementary file 2 — Figure S2. Correlation between CLEC3B expression and clinicopathological characteristic, and improvement of the TNM staging prognostic model with CLEC3B expression. (A) Receiver operating characteristic (ROC) curve analyses of different cutoff values of composite expression score (CES), and the area under the ROC curve (AUC), 95% confident interval (95% CI) and P-value are shown. (B, C) The disease free time in IHC staining (n = 80, P < 0.0001) (B) and TCGA-LIHC database (n = 315, P < 0.0001) (C), based on CLEC3B expression level, were calculated by Kaplan–Meier. (D) The relative proportion of patients with low CLEC3B expression is increased with the tumor progression in hepatocellular carcinoma (P = 0.006). (E) Multivariate Cox analysis was conducted to analyze independent prognostic factors in patients with hepatocellular carcinoma. (F) ROC analysis of the sensitivity and specificity for the predictive value of CLEC3B expression model, TNM model and the combined model of CLEC3B and TNM. (G) AIC and C-index, another prognostic predicting model nomogram for overall survival, were performed to analyze the predictive accuracies of TNM stage, CLEC3B expression and the combined model of CLEC3B and TNM. (H) Nomogram was built to quantify the combined effect of the proven independent prognostic factors for overall survival. (I) Calibration plot of the nomogram for 5-year survival. (J) Of all patients, three groups were divided according to the total points in the nomogram which range of 0–40, 41–120, 121–160, was refined as low risk, medium and high risk subgroup (P < 0.0001). Kaplan–Meier analysis was used to test the correlation of the risk with overall survival. *, P < 0.05; **, P < 0.01; ***, P < 0.001; n.s., not significant. (TIF 827 kb) [file 12964_2019_423_MOESM2_ESM.tif]

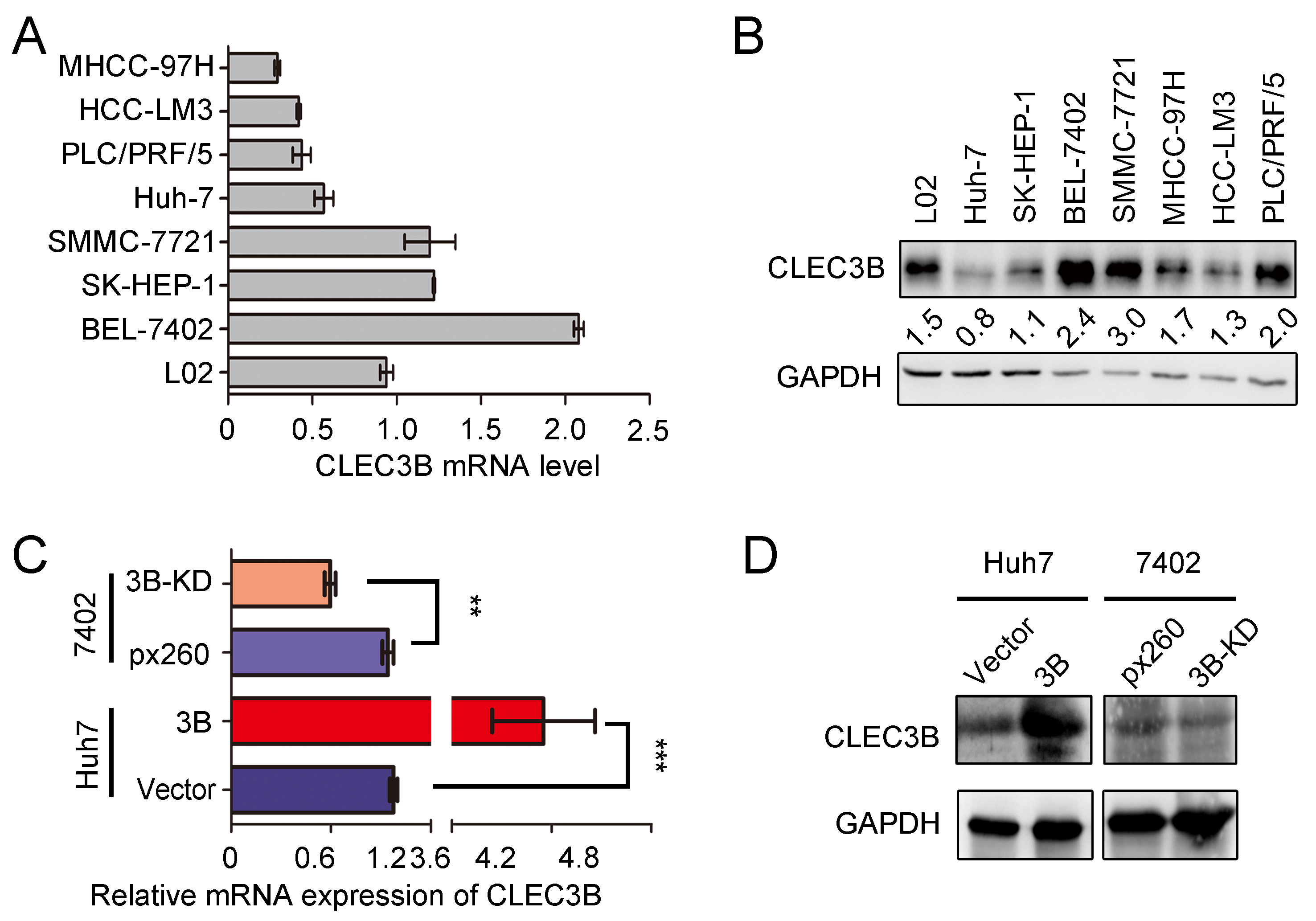

Supplement: Supplementary file 5 — Figure S3. CLEC3B was significantly decreased in HCC cells. (A, B) The mRNA and protein level of CLEC3B in different hepatocellular carcinoma cell lines were analyzed by real-time polymerase chain reaction (RT-PCR) (A) and western blot (B). (C) The overexpression (P = 0.0005) and knockdown (P = 0.0018) efficiency of relative mRNA expression of plasmids in HCC cells. (D) The overexpression and knockdown efficiency of protein expression of plasmids in HCC cells. *, P < 0.05; **, P < 0.01; ***, P < 0.001; n.s., not significant. (TIF 657 kb) [file 12964_2019_423_MOESM5_ESM.tif]

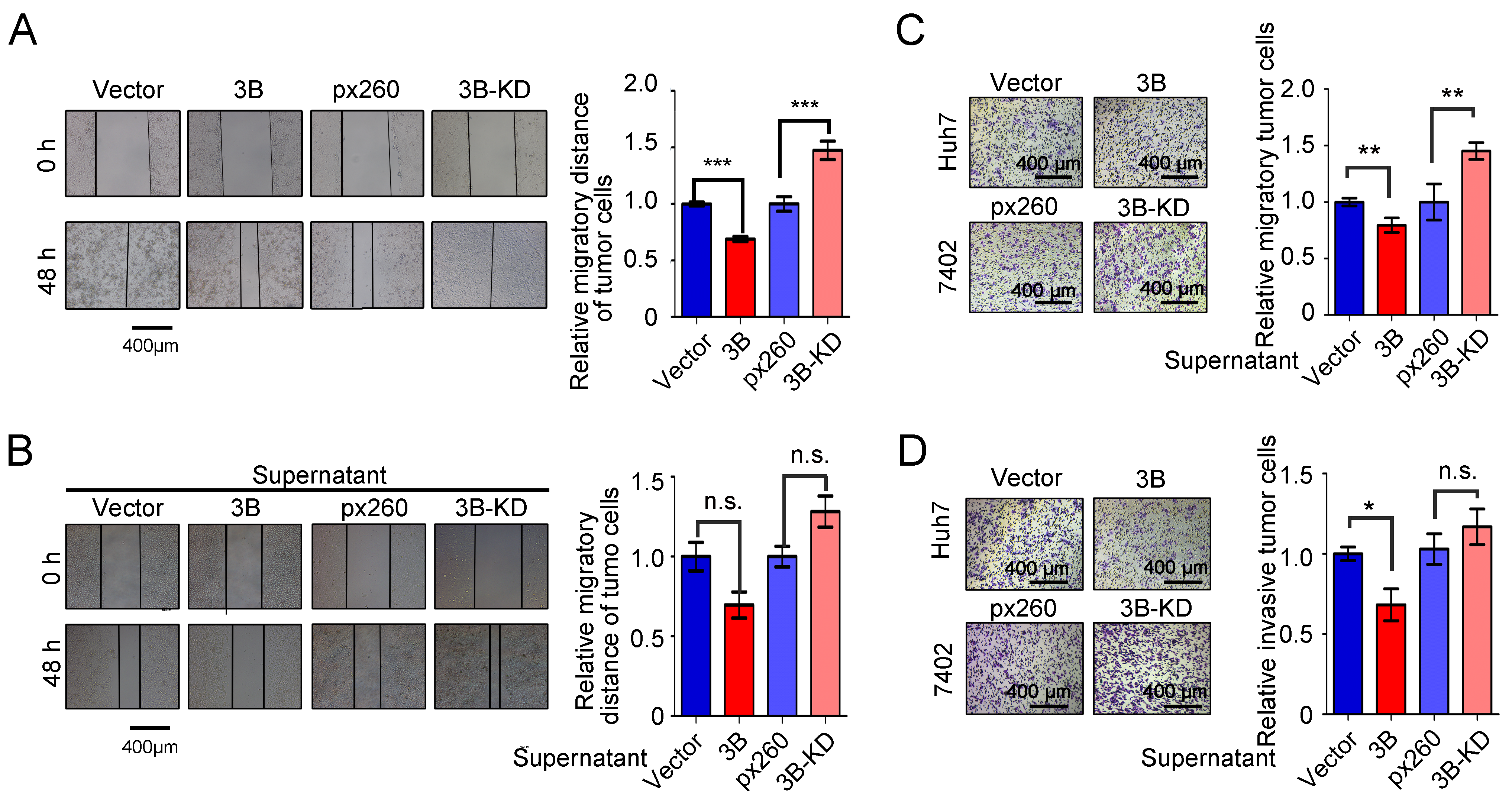

Supplement: Supplementary file 6 — Figure S4. Down-regulated CLEC3B in HCC promoted migration and invasion of HCC cells via supernatant. (A) Representative images and statistical data of migratory distance of HCC cells with CLEC3B overexpression (3B, P < 0.0001) or CLEC3B knockdown (3B-KD, P < 0.0001) using wound healing assay. (B) Representative images and migratory distance of HCC cells treated with the supernatant from HCC cells with CLEC3B overexpression (3B; P = 0.0603) or down-regulated (3B-KD; P = 0.0572) in wound healing assays. (C) Representative images and migratory number of HCC cells treated with supernatant from 3B (P = 0.009) or 3B-KD (P = 0.0087) tumor cells using transwell assays. (D) Representative images and invasive number of HCC cells treated with supernatant from 3B (P = 0.015) or 3B-KD (P = 0.3806) tumor cells using invasive assays. *, P < 0.05; **, P < 0.01; ***, P < 0.001; n.s., not significant. (TIF 4362 kb) [file 12964_2019_423_MOESM6_ESM.tif]

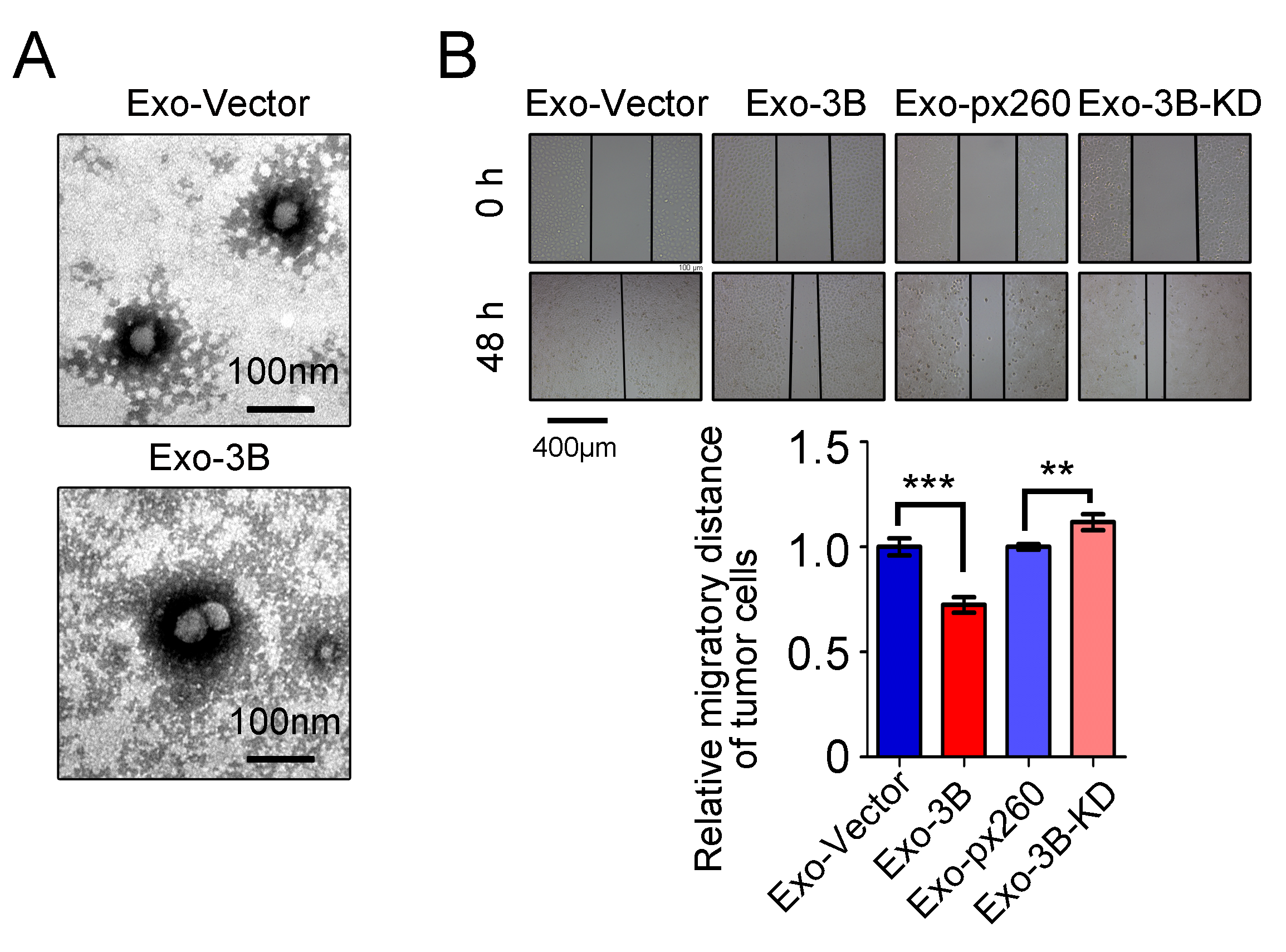

Supplement: Supplementary file 7 — Figure S5. CLEC3B was significantly down-regulated in exosomes derived from HCC. (A) Representative micrograph of exosomes derived from 3B cells. (B) Representative images and statistical data of migratory distance of HCC cells treated with exosomes from HCC cells with 3B (Exo-3B, P = 0.0001) or 3B-KD (Exo-3B-KD, P = 0.0079) using wound healing assay. *, P < 0.05; **, P < 0.01; ***, P < 0.001; n.s., not significant. (TIF 1851 kb) [file 12964_2019_423_MOESM7_ESM.tif]

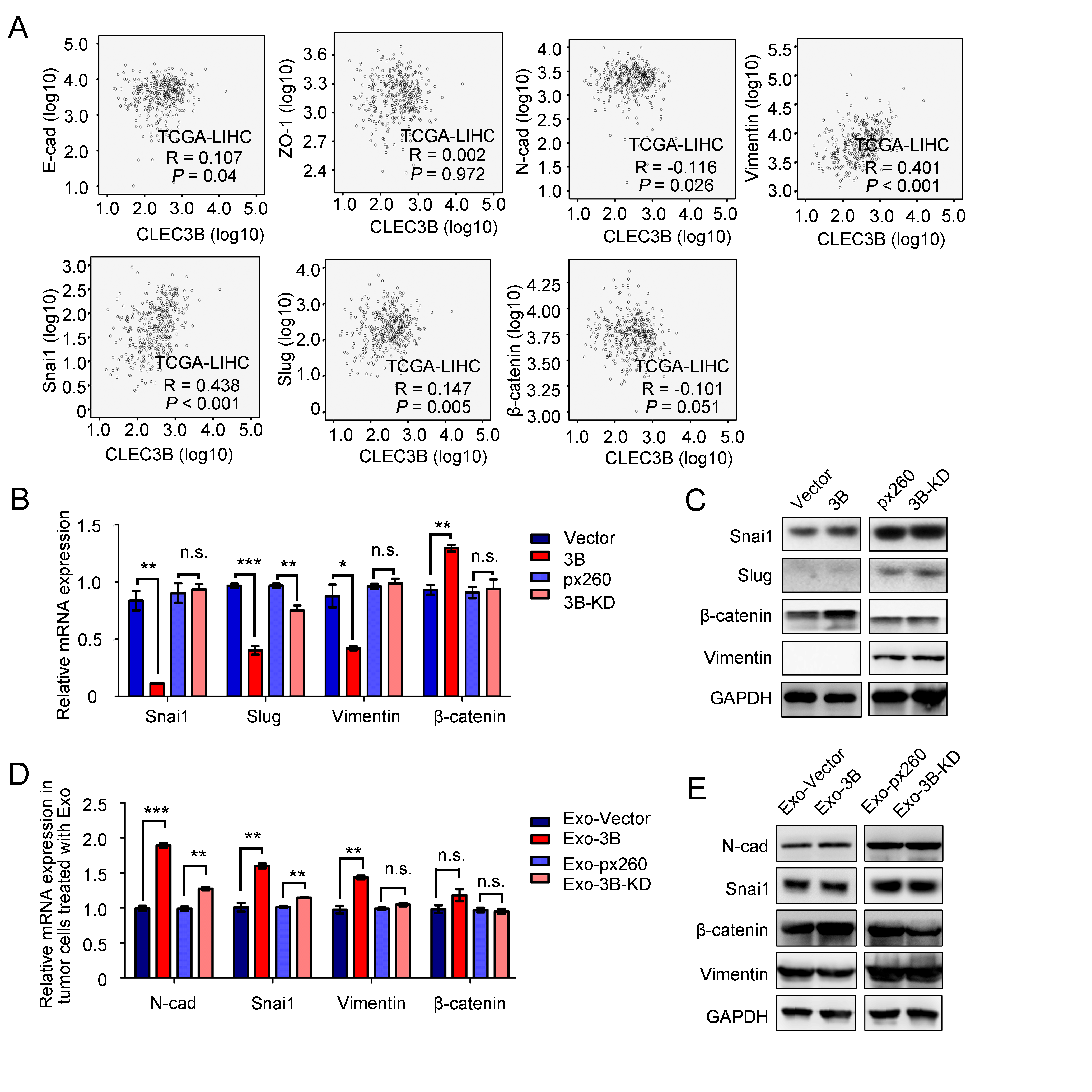

Supplement: Supplementary file 8 — Figure S6. Exosomal CLEC3B inhibited EMT of HCC cells. (A) Analysis of correlation of CLEC3B with E-cad (R = 0.107, P = 0.04), ZO-1 (R = 0.002, P = 0.972), N-cad (R = − 0.116, P = 0.026), Snai1 (R = 0.438, P < 0.001), Slug (R = 0.147, P = 0.005),β-catenin (R = − 0.101, P = 0.051) and Vimentin (R = 0.401, P < 0.001) in TCGA-LIHC database. (B) The relative mRNA expression of EMT relative molecules in HCC cells transfected with 3B (Snai1, P = 0.0010; Slug, P = 0.0002; Vimentin, P = 0.0107; β-catenin, P = 0.0023) or 3B-KD (Snai1, P = 0.7509; Slug, P = 0.0100; Vimentin, P = 0.6157; β-catenin, P = 0.7604) plasmids. (C) The protein expression of EMT relative molecules in HCC cells transfected with 3B or 3B-KD plasmids. (D) The mRNA expression of N-cad (Exo-3B, P < 0.0001; Exo-3B-KD, P = 0.0015), Snai1 (Exo-3B, P = 0.0011; Exo-3B-KD, P = 0.0010), β-catenin (Exo-3B, P = 0.0015; Exo-3B-KD, P = 0.1158) and Vimentin (Exo-3B, P = 0.1211; Exo-3B-KD, P = 0.7113) in tumor cells, which were treated with exosomes. (E) Levels of protein related to EMT in tumor cells treated with Exo-3B or Exo-3B-KD. *, P < 0.05; **, P < 0.01; ***, P < 0.001; n.s., not significant. (TIF 1974 kb) [file 12964_2019_423_MOESM8_ESM.tif]

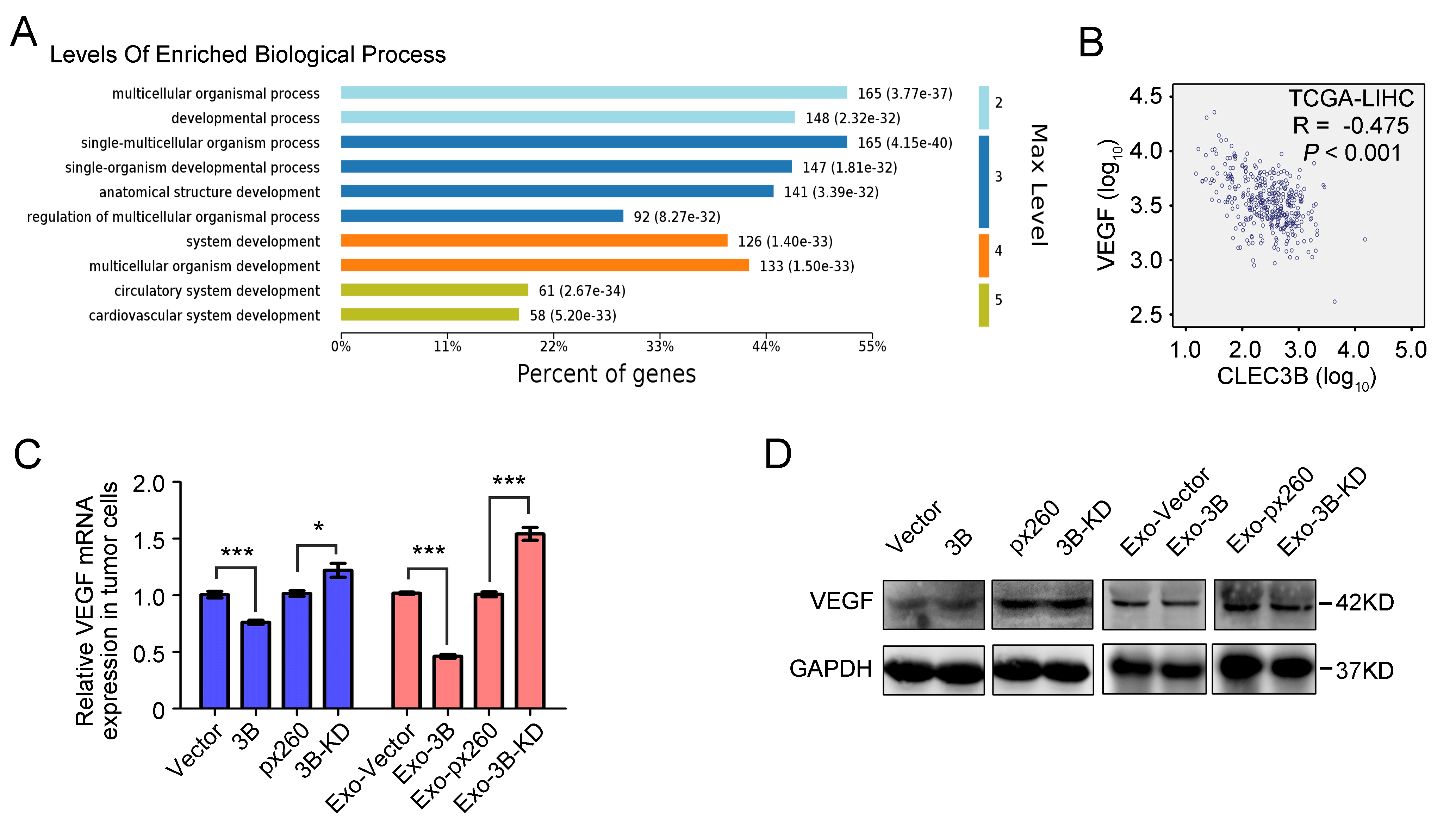

Supplement: Supplementary file 9 — Figure S7. Exosomal CLEC3B decreased VEGF in HCC cells to inhibit angiogenesis. (A) Enriched biological process of genes significantly correlated with CLEC3B in TCGA database. (B) Correlation between CLEC3B and VEGF (Pearson, R = − 0.475, P < 0.001) in TCGA-LIHC database. (C) Relative mRNA expression of VEGF in HCC cells with 3B (P < 0.001) or 3B-KD (P = 0.0183), and treated with Exo-3B (P < 0.0001) or 3B-KD (P = 0.0009). (D) Protein levels of VEGF in 3B or 3B-KD HCC cells or HCC cells treated with Exo-3B-treated or Exo-3B-KD. *, P < 0.05; **, P < 0.01; ***, P < 0.001; n.s, not significant. (TIF 973 kb) [file 12964_2019_423_MOESM9_ESM.tif]

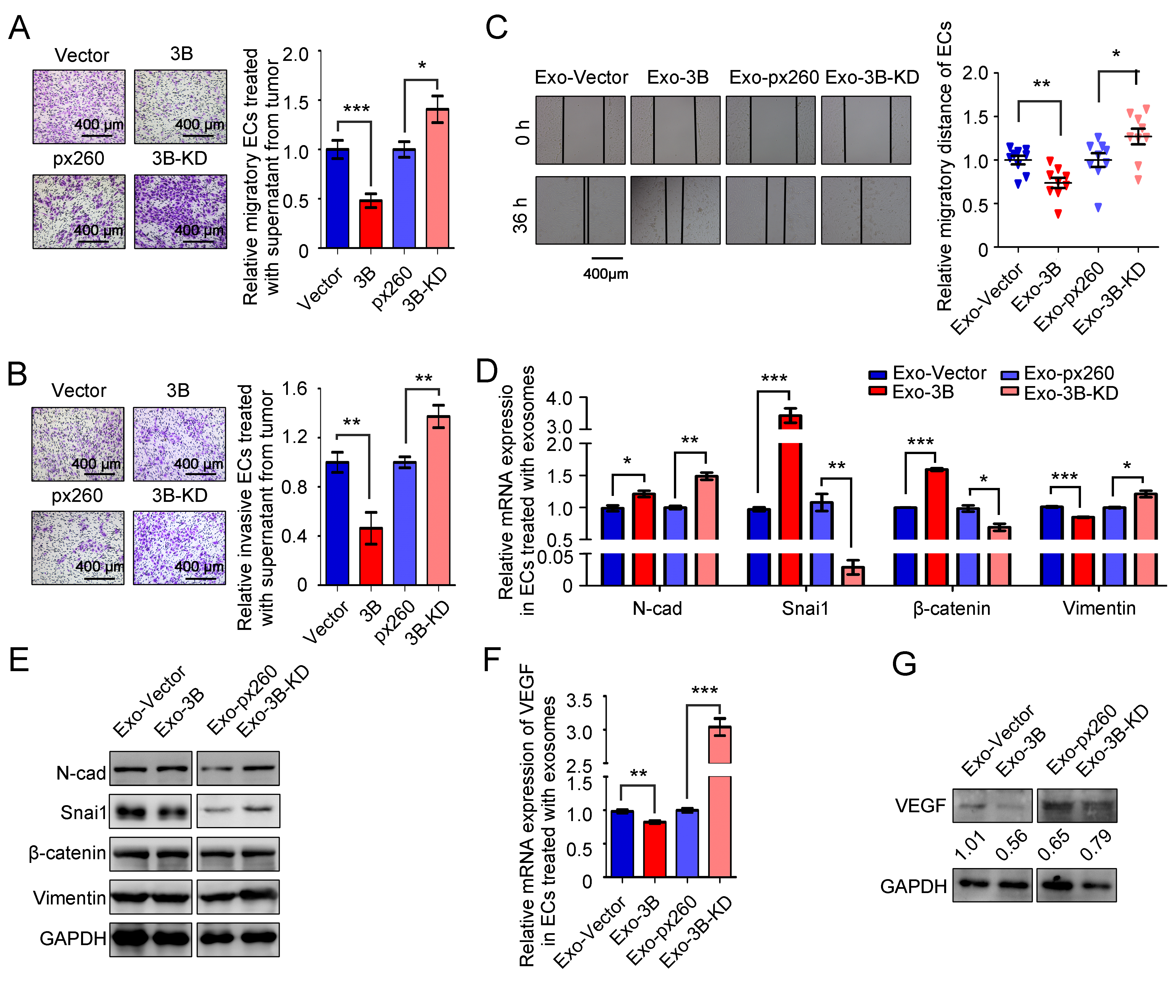

Supplement: Supplementary file 11 — Figure S8. Exosomal CLEC3B inhibited migration, invasion and EMT of ECs. (A) Representative images and relative migratory number of ECs treated with supernatant from 3B (P = 0.0003) or 3B-KD (P = 0.0191) HCC tumor cells in transwell assays. (B) Representative images and relative invasive number of ECs treated with supernatant from 3B (P = 0.0029) or 3B-KD (P = 0.0011) tumor cells in invasive assays. (C) Representative images and relative migratory distance of ECs treated with Exo-3B (P = 0.0033) or Exo-3B-KD (P = 0.0377) in wound healing assays. (D) The relative mRNA expression of N-cad (Exo-3B, P = 0.0287; Exo-3B-KD, P = 0.0014), Snai1 (Exo-3B, P = 0.0004; Exo-3B-KD, P = 0.0014), β-catenin (Exo-3B, P < 0.0001; Exo-3B-KD, P = 0.0166) and Vimentin (Exo-3B, P = 0.0003; Exo-3B-KD, P = 0.0139) in ECs treated with exosomes from tumor cells. (E) The protein level of N-cad, Snai1, β-catenin and Vimentin in ECs treated with Exo-3B or Exo-3B-KD from tumor cells. (F) Relative VEGF mRNA expression in ECs treated with exosomes (Exo-3B, P = 0.0075; Exo-3B-KD, P = 0.0001). (G) Protein level of VEGF in ECs treated with exosomes. *, P < 0.05; **, P < 0.01; ***, P < 0.001; n.s., not significant. (TIF 3752 kb) [file 12964_2019_423_MOESM11_ESM.tif]

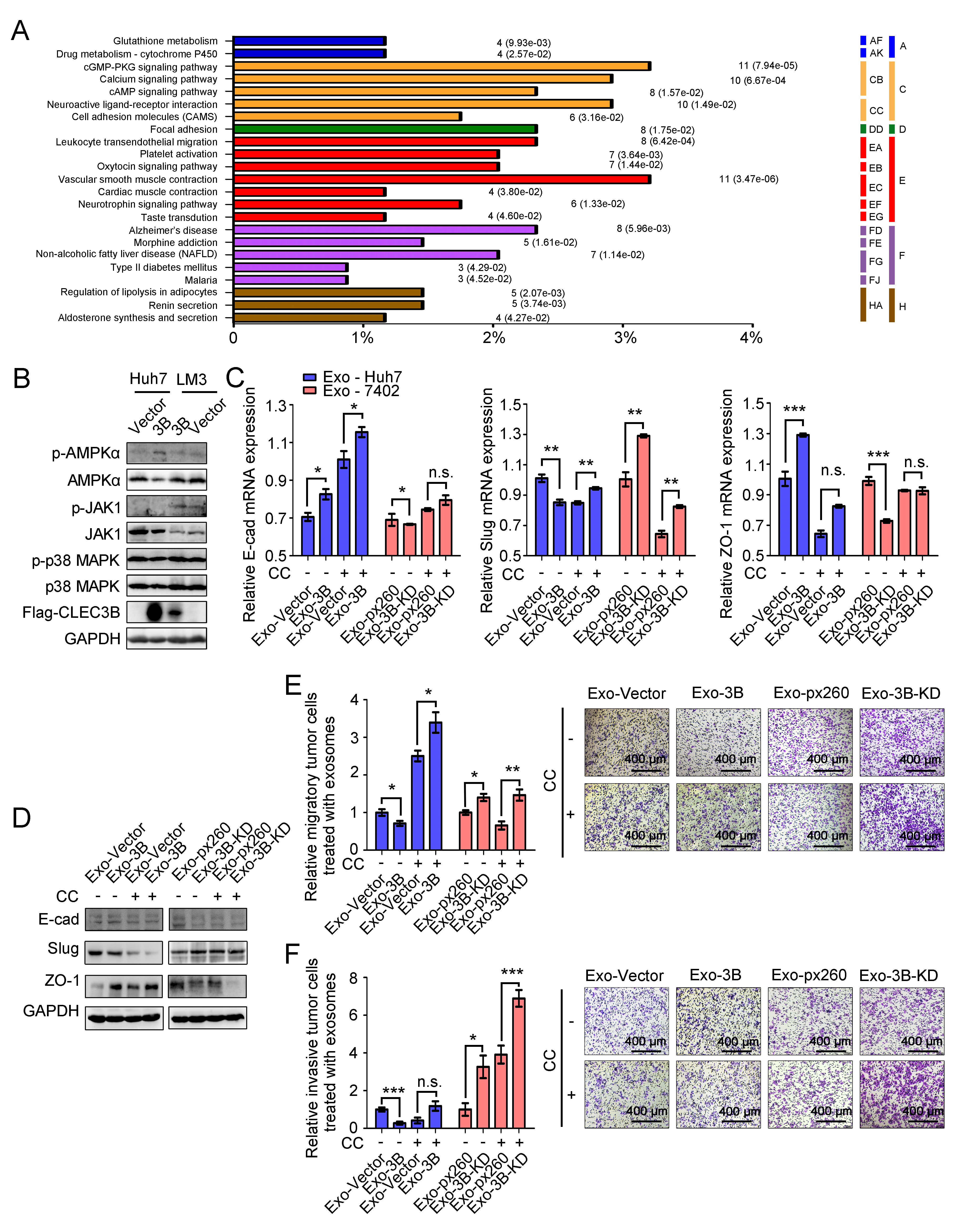

Supplement: Supplementary file 12 — Figure S9. Exosomal CLEC3B inhibited migration, invasion and EMT independent of AMPK signaling pathway in HCC cells. (A) KEGG enrichment CLEC3B-correlated genes. A: Metabolism; AF: metabolism of other amino acids; AK: Xenobiotics biodegradation and metabolism; C: Environmental Information Processing; CB: Signaling transduction; CC: Signaling molecules and interaction; D: Cellular Processes; DD: Cellular community; E: Organismal Systems; EA: Immune system; EB: Endocrine system; EC: Circulatory system; EF: Nervous system; EG: Sensory system; F: Human Diseases; FD: Neurodegenerative diseases; FE: Substance dependence; FG: Endocrine and metabolic diseases; H: Other and unknown; HA: Other and unknown. (B) Expression of proteins in tumor cells affected by CLEC3B. (C) Relative mRNA expression of E-cad (Exo-3B, no CC, P = 0.0266; CC, P = 0.0491; Exo-3B-KD, no CC, P = 0.0473; CC, P = 0.1337), Slug (Exo-3B, P = 0.0069, P = 0.0019; Exo-3B-KD, P = 0.0040, P = 0.0016) and ZO-1 (Exo-3B, P < 0.0001, P = 0.0016; Exo-3B-KD, P = 0.0008, P = 0.9323) in tumor cells treated with Compound C (CC, a drug to inhibit phosphorylation of AMPK) and EXO-3B or Exo-3B-KD. (D) Protein expression of E-cad, Slug and ZO-1 in tumor cells treated with CC and EXO-3B or Exo-3B-KD. (E) Representative images and relative migratory number of tumor cells treated with CC or not and Exo-3B (no CC, P = 0.0401; CC, P = 0.0165) or Exo-3B-KD (no CC, P = 0.0140; CC P = 0.0024). (F) Representative images and relative invasive number of tumor cells treated with CC or not and Exo-3B (no CC, P = 0.0003; CC, P = 0.0618) or Exo-3B-KD (no CC, P = 0.0409; CC, P = 0.0005). *, P < 0.05; **, P < 0.01; ***, P < 0.001; n.s., not significant. (TIF 5213 kb) [file 12964_2019_423_MOESM12_ESM.tif]

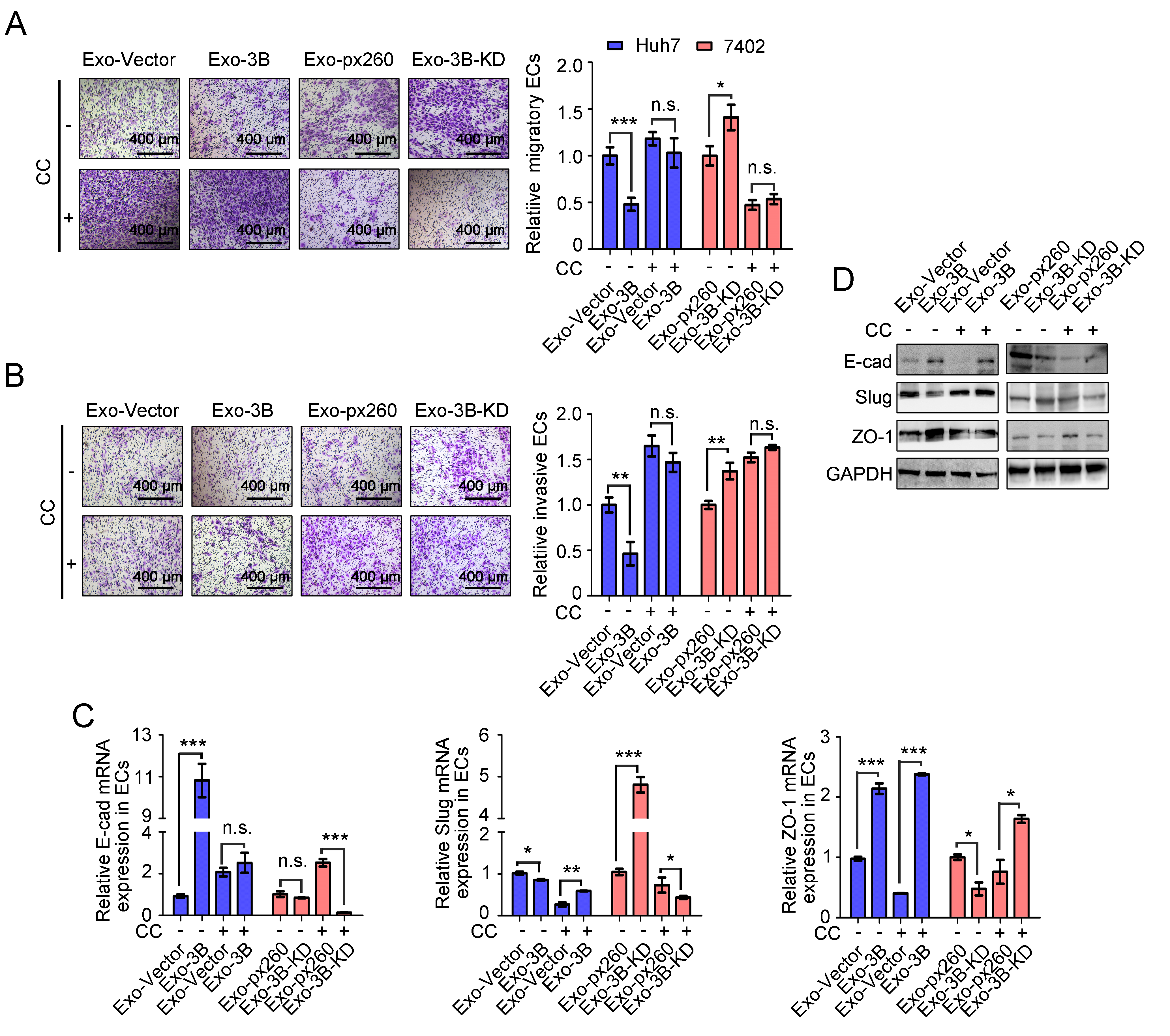

Supplement: Supplementary file 14 — Figure S10. Exosomal CLEC3B inhibiting migration, invasion and EMT were AMPK signaling-independent in ECs. (A) Representative images and relative migratory number of ECs incubated with CC and exosomes from tumor cells, Exo-3B (no CC, P = 0.0003; CC, P = 0.3142) and Exo-3B-KD (no CC, P = 0.0389; CC, P = 0.4269). (B) Representative images and relative invasive number of ECs incubated with CC and Exo-3B (no CC, P = 0.0029; CC, P = 0.2830) or Exo-3B-KD (no CC, P = 0.0011; CC, P = 0.0733). (C) Relative mRNA expression of E-cad (Exo-3B, no CC, P = 0.0002; CC, P = 0.4442; Exo-3B-KD, no CC, P = 0.0509; CC, P = 0.0002), Slug (Exo-3B, P = 0.0159, P = 0.0030; Exo-3B-KD, no CC, P < 0.0001; CC, P = 0.0920) and ZO-1 (Exo-3B, no CC, P = 0.0002; CC, P < 0.0001; Exo-3B-KD, no CC, P = 0.0110; CC, P = 0.0134) in ECs treated with CC and Exo-3B or Exo-3B-KD. (D) Expression of E-cad, Slug and ZO-1 in ECs treated with CC and Exo-3B or Exo-3B-KD. *, P < 0.05; **, P < 0.01; ***, P < 0.001; n.s., not significant. (TIF 4765 kb) [file 12964_2019_423_MOESM14_ESM.tif]
