# Supplementary material for: Downregulation of exosomal CLEC3B in hepatocellular carcinoma promotes metastasis and angiogenesis via AMPK and VEGF signals
Source: Cell Commun Signal. 2019 Sep 2;17:113. doi: 10.1186/s12964-019-0423-6 (PMC6721425; doi:10.1186/s12964-019-0423-6)
Supplement: Supplementary file 3 — Table S1. CLEC3B expression and relative factors. (DOCX 15 kb) [file 12964_2019_423_MOESM3_ESM.docx]

| **Variables** | **patients (N=80)** | **CLEC3B expression** | |  |
| --- | --- | --- | --- | --- |
|  |  | **High**  **No. (%)** | **Low**  **No. (%)** | ***P*-value** |
| **Age (y)** | | | | |
| = < 50 | 40 | 14 (35.0) | 26 (65.0) | 0.409 |
| > 50 | 40 | 16 (40.0) | 24 (60.0) |  |
| **Gender** | | | | |
| Male | 68 | 24 (35.3) | 44 (64.7) | 0.256 |
| Female | 12 | 6 (50.0) | 6 (50.0) |  |
| **Tumor size (cm)** | | | | |
| <5 | 39 | 22 (56.4) | 17 (43.6) | 0.001 |
| >=5 | 41 | 8 (19.5) | 33 (80.5) |  |
| **TNM stage** | | | | |
| I + II | 40 | 22 (55.0) | 18 (45.0) | 0.002 |
| III + IV | 40 | 8 (20.0) | 32 (80.0) |  |
| **Vessel metastasis** | | | | |
| Negative | 48 | 24 (50.0) | 24 (50.0) | 0.004 |
| Positive | 32 | 6 (18.8) | 26 (81.2) |  |
| **Depth of invasion** | | | | |
| T1 + T2 | 43 | 24 (55.8) | 19 (44.2) | <0.001 |
| T3 + T4 | 37 | 6 (16.2) | 31 (83.8) |  |
| **Lymph node metastasis** | | | | |
| Negative | 77 | 29 (37.7) | 48 (62.3) | 0.686 |
| Positive | 3 | 1 (33.3) | 2 (66.7) |  |
| **Distant metastasis** | | | | |
| Negative (M0) | 72 | 28 (38.9) | 44 (61.1) | 0.360 |
| Positive (M1) | 8 | 2 (25.0) | 6 (75.0) |  |

Additional file 3: **Table S1. Relationship between TN expression and clinicopathological characteristics in patients with hepatocellular carcinoma.** TNM = tumor node metastasis. *χ 2 test, Fisher exact test or Student’s t test was performed. *P*-value < 0.05 shows statistical significant.
