# Supplementary material for: Downregulation of exosomal CLEC3B in hepatocellular carcinoma promotes metastasis and angiogenesis via AMPK and VEGF signals
Source: Cell Commun Signal. 2019 Sep 2;17:113. doi: 10.1186/s12964-019-0423-6 (PMC6721425; doi:10.1186/s12964-019-0423-6)
Supplement: Supplementary file 4 — Table S2. Survival time of HCC patients and relative factors. (DOCX 16 kb) [file 12964_2019_423_MOESM4_ESM.docx]

| **variables** | **Univariate** | | | **Multivariate** | | |
| --- | --- | --- | --- | --- | --- | --- |
|  | **HR** | **95% CI** | ***P* - value** | **HR** | **95% CI** | ***P* - value** |
| Age (years) | | | | | | |
| = < 50 vs > 50 | 0.653 | 0.373-1.144 | 0.136 |  |  |  |
| Gender | | | | | | |
| Male vs Female | 0.449 | 0.198-1.259 | 0.141 |  |  |  |
| Lymph node metastasis | | | | | | |
| Negative vs Positive | 7.663 | 2.251-26.085 | 0.001 |  |  |  |
| Tumor size cm | | | | | | |
| <5 vs >=5 | 3.016 | 1.668-5.453 | 0.141 | 1.64 | 0.88-3.06 | 0.122 |
| Vessel invasion | | | | | | |
| Negative vs Positive | 3.794 | 2.146-6.706 | <0.001 | 1.01 | 0.43-2.35 | 0.987 |
| Tumor invasion depth | | | | | | |
| I+II vs III+IV | 5.247 | 2.852-9.652 | <0.001 | 1.18 | 0.54-2.54 | 0.628 |
| Distant metastasis | | | | | | |
| Negative (M0) vs  Positive (M1) | 3.301 | 1.513-7.204 | 0.003 | 0.42 | 0.08-2.08 | 0.286 |
| TN expression | | | | | | |
| Low vs high | 3.707 | 1.885-7.288 | <0.001 | 2.74 | 1.34-5.59 | 0.006 |
| TNM stage | | | | | | |
| I+II vs III+IV | 6.361 | 3.328-12.155 | <0.001 | 3.74 | 1.21-10.02 | 0.021 |

Additional file 4: Table S2. **Univariate Cox regression analysis of clinicopathological characteristics influencing the overall survival of 80 pairs of patients with hepatocellular carcinoma.** 95% CI = 95% confidence interval; HR = hazard ratio; TNM = tumor node metastasis. *P*-value < 0.05 shows statistically significant.
